# Predicting the potential distribution of four endangered holoparasites and their primary hosts in China under climate change

**DOI:** 10.3389/fpls.2022.942448

**Published:** 2022-08-03

**Authors:** Xin Lu, Ruoyan Jiang, Guangfu Zhang

**Affiliations:** Jiangsu Key Laboratory of Biodiversity and Biotechnology, School of Life Sciences, Nanjing Normal University, Nanjing, China

**Keywords:** MaxEnt, niche overlap, parasitic plants, suitable habitat, conservation

## Abstract

Climate change affects parasitic plants and their hosts on distributions. However, little is known about how parasites and their hosts shift in distribution, and niche overlap in response to global change remains unclear to date. Here, the potential distribution and habitat suitability of four endangered holoparasites and their primary hosts in northern China were predicted using MaxEnt based on occurrence records and bioclimatic variables. The results indicated that (1) Temperature annual range (Bio7) and Precipitation of driest quarter (Bio17) were identified as the common key climatic factors influencing distribution (percentage contribution > 10%) for *Cynomorium songaricum* vs. *Nitraria sibirica* (i.e., parasite vs. host); Temperature seasonality (Bio4) and Precipitation of driest month (Bio14) for *Boschniakia rossica* vs. *Alnus mandshurica*; Bio4 for *Cistanche deserticola* vs. *Haloxylon ammodendron*; Precipitation of warmest quarter (Bio18) for *Cistanche mongolica* vs. *Tamarix ramosissima*. Accordingly, different parasite-host pairs share to varying degree the common climatic factors. (2) Currently, these holoparasites had small suitable habitats (i.e., moderately and highly) (0.97–3.77%), with few highly suitable habitats (0.19–0.81%). Under future scenarios, their suitable habitats would change to some extent; their distribution shifts fell into two categories: growing type (*Boschniakia rossica* and *Cistanche mongolica*) and fluctuating type (*Cynomorium songaricum* and *Cistanche deserticola*). In contrast, the hosts’ current suitable habitats (1.42–13.43%) varied greatly, with highly restricted suitable habitats (0.18–1.00%). Under future scenarios, their suitable habitats presented different trends: growing type (*Nitraria sibirica*), declining type (*Haloxylon ammodendron*) and fluctuating type (the other hosts). (3) The niche overlaps between parasites and hosts differed significantly in the future, which can be grouped into two categories: growing type (*Boschniakia rossica* vs. *Alnus mandshurica*, *Cistanche mongolica* vs. *Tamarix ramosissima*), and fluctuating type (the others). Such niche overlap asynchronies may result in severe spatial limitations of parasites under future climate conditions. Our findings indicate that climate factors restricting parasites and hosts’ distributions, niche overlaps between them, together with parasitic species identity, may jointly influence the suitable habitats of parasitic plants. Therefore, it is necessary to take into account the threatened holoparasites themselves in conjunction with their suitable habitats and the parasite-host association when developing conservation planning in the future.

## Introduction

Parasitic plants derive water and nutrients from other organisms through haustoria. There are approximately 4,750 species of parasitic plants worldwide, and they occur in almost every biome (Těšitel, 2016; Nickrent, 2020). Although some of them are harmful to forestry or agriculture, resulting in reduced production or crop losses, these parasitic plants play a significant role in different ecosystems (Jiang and Zhang, 2021). Moreover, in some regions, parasitic plants are used as traditional medicinal herbs. According to the dependence on the host for nutrients, parasitic plants can be classified into hemiparasites and holoparasites.

Climate change can alter the biotic and abiotic environment of plant species, and accordingly affect their geographic distributions (Bellard et al., 2012; Jarvie and Svenning, 2018; Gomes et al., 2020). In the case of parasitic plants, climate change affects their growth not only directly but also indirectly by affecting their host plants. Phoenix and Press (2005) predicted that climate change, such as elevated carbon dioxide concentration and rising temperature, would promote photosynthesis of the root hemiparasites from Orobanchaceae and their hosts and that it would simultaneously increase the mineral nutrient requirements of hosts, thus regulating the host-parasite association. Compared with hemiparasitic plants, holoparasitic plants depend completely on their hosts for survival, with higher host specificity (Andrea and Sergi, 2021). As a result, holoparasites are more closely associated with their hosts relative to hemiparasites. Therefore, climate change may have more significant impact on the association of holoparasite-host than of hemiparasite-host. Fontúrbel et al. (2021) contended that climate change would alter and disrupt ecological interactions, resulting in complex cascade effects and thus affecting biodiversity at the community level.

Theoretically, for both hemiparasites and holoparasites, climate change affects not only their own but also their hosts’ distribution (Mkala et al., 2022). However, compared with autophytes, there are very few studies available so far about the impact of climate change on the distribution of parasitic plants. Zamora and Mellado (2019) explored the key driving factors influencing the distribution shift of *Viscum album* subsp. *austriacum*, a hemiparasitic shrub. Namely, it migrated to the summit along the elevational gradient in the Mediterranean mountains with warming temperature. Wang et al. (2019) pointed out that the occupied habitat of *Pedicularis kansuensis*, a root hemiparasitic herb, would shift northward in China under climate change scenarios. Liu et al. (2019) evaluated the habitat suitability of *Cistanche deserticola*, a holoparasitic perennial, in northwest China under future climate scenarios (2050s, 2070s). Ren et al. (2020) predicted the potential distribution of *Cuscuta chinensis*, a stem holoparasitic vine, under global warming. These studies have addressed the projected distribution shift and expanding invasion areas of parasitic plants under a global-change scenario. However, we notice that few parasitic species are involved in such studies and even a single parasite for most studies. Recently, some researchers have taken to studying the distribution prediction of a host plant. Chang et al. (2019) used three ecological niche models to predict the potential distribution of *Haloxylon ammodendron*, which is a primary host plant of parasitic *Cistache deserticola*, in the arid area of northwest China under future climate change. They considered that under RCP4.5 and RCP8.5 its total suitable distribution area would increase over time, especially the highly suitable distribution area. Therefore, most studies have focused on the distribution prediction of parasites or hosts, but unfortunately, few studies consider the influence of climate change on the relationship between parasites and hosts.

More recently, only a small number of studies have taken into account the relationship between parasitic plants and hosts when analyzing the impact of climate change on parasites. Kukushkin et al. (2017) accounted for the limited distribution of hemiparasitic *Arceuthobium oxycedri* in the low mountain areas of Crimea by GIS techniques, which may be related to the formation of its host *Juniperus deltoides* range in the late Pleistocene-Holocene and a low speed of the hemiparasite dissemination from Quaternary refugia in the Crimean Peninsula. Renjana et al. (2022) predicted the suitable potential habitat of the parasitic *Rafflesia arnoldii* by studying the potential distribution of its host plants through MaxEnt model. Indeed, several recent studies have considered the impact of future climate change on both parasites and host plants. Mkala et al. (2022) used five models to analyze the impact of climate change on the distribution of *Hydnora abyssinica*, *H. africana* and their hosts. He et al. (2021) projected the suitable habitats of the desert parasite based on an ecological niche model, using the parasitic *Cistanche deserticola* and its host *Haloxylon ammodendron* as examples. However, such an approach of taking the occurrence record from both a parasite and its host as that from one single ‘virtual’ species is questionable in this study, because it seems unlikely to reflect the close link between parasites and hosts. In fact, it should be noted that ecological niche overlap has recently been used to characterize the degree of similarity of distribution for closely related species pairs under climate change (Filz and Schmitt, 2015; Yin et al., 2021; Jiang et al., 2022).

Ecological (or Environmental) niche models (ENMs), also known as species distribution models (SDMs), are numerical tools that use species occurrence records in conjunction with environmental conditions to infer the niches of species and their habitat suitability according to specific algorithms (Elith and Leathwick, 2009). At present, the main ENMs and software include BIOCLIM, BIOMOD, CLIMEX, DivaGIS, DOMAIN, genetic algorithm for rule-set prediction (GARP), and MaxEnt (Phillips and Dudík, 2008; Ahmed et al., 2015). Among them, MaxEnt model is one of the most widely used methods because of its ease of use, superior performance, small sample size requirement, flexibility of variable processing, and good noise reduction effect (Phillips et al., 2006; Merow et al., 2013; Sillero and Barbosa, 2021). Therefore, the MaxEnt model has been extensively applied in forecasting the possible distribution of species in the future.

In this study, we used MaxEnt model to predict the distribution of four endangered holoparasitic plants and their primary hosts in northern China under current and future climate scenarios. More specifically, we aimed to (1) identify the key climatic factors affecting the suitable habitat of four parasitic plants and their primary hosts, and the common major climatic factors of each parasite-host pair as well; (2) predict the suitable habitats of the four parasitic plants and their primary hosts under current and future climate scenarios; (3) measure the ecological niche overlaps between parasites and hosts under different climate scenarios and analyze the variation of niche overlap of each parasite-host pair. Moreover, we further determined the basic features of suitable habitat changes for the four species pairs under future climate scenarios, and presented the main reasons for these changes. The purpose of this study is to shed light on the mechanisms of holoparasites together with their primary hosts in response to climate change, and to provide useful information for the conservation and management of parasitic plants.

## Materials and methods

### Species selection and occurrence records

**FIGURE 1:**
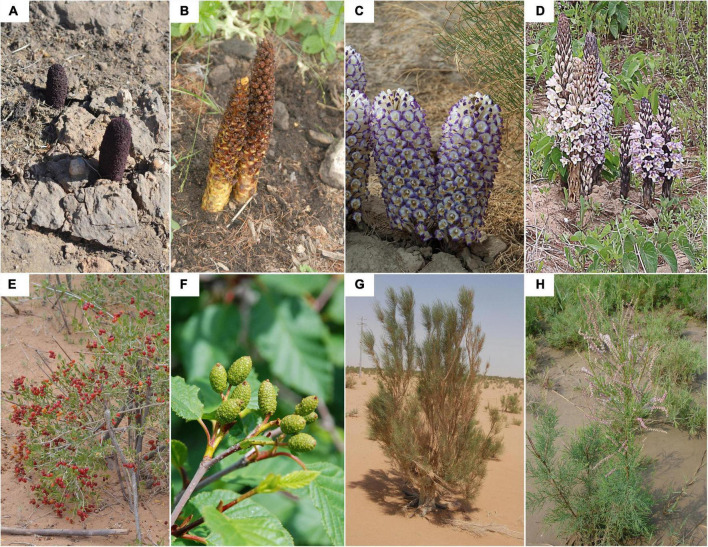
The habitat and morphological features of four holoparasitic plants **(A–D)** and their primary hosts **(E–H)**. **(A)** *Cynomorium songaricum*; **(B)** *Boschniakia rossica*; **(C)** *Cistanche deserticola*; **(D)** *Cistanche mongolica*; **(E)** *Nitraria sibirica*; **(F)** *Alnus mandshurica*; **(G)** *Haloxylon ammodendron*; **(H)** *Tamarix ramosissima*. The photographs **(A,B,E)** were provided by Liu L.; **(C,D**,**G)** by Duan S., Zang D., and Zhao D. respectively; **(F,H)** by Zhang G.

There are 745 parasitic angiosperms in China, in which 66 species are root holoparasites (Jiang and Zhang, 2021). Four of them have been ranked the second class on *the List of National Key Protected Wild Plants in China* since September of 2021 (State Forestry and Grassland Administration and the Ministry of Agriculture and Rural Affairs, P. R. China [SFGA], 2021). In light of *the IUCN Red List Category and Criteria*, there is one species (i.e., *Cistanche deserticola*) of Endangered (EN), three Vulnerable (VU) (Qin et al., 2017). Accordingly, the four species are endangered holoparasitic angiosperms. In addition, they are mainly distributed in the northern part of China (Wu et al., 1998, 2007). Therefore, we selected the four species, namely *Cynomorium songaricum* Rupr., *Boschniakia rossica* (Chamisso et Schlechtendal) B. Fedtschenko, *Cistanche deserticola* Ma, and *Cistanche mongolica* Beck, in the current study. Meanwhile, based on our field investigation (Figure 1) and related references (Wu et al., 1998; Ren et al., 2018; He et al., 2021), we then selected *Nitraria sibirica* Pall., *Alnus mandshurica* (Callier ex C. K. Schneider) Hand.-Mazz., *Haloxylon ammodendron* (C. A. Mey.) Bunge, and *Tamarix ramosissima* Ledeb. as their primary host plants, respectively.

**FIGURE 2:**
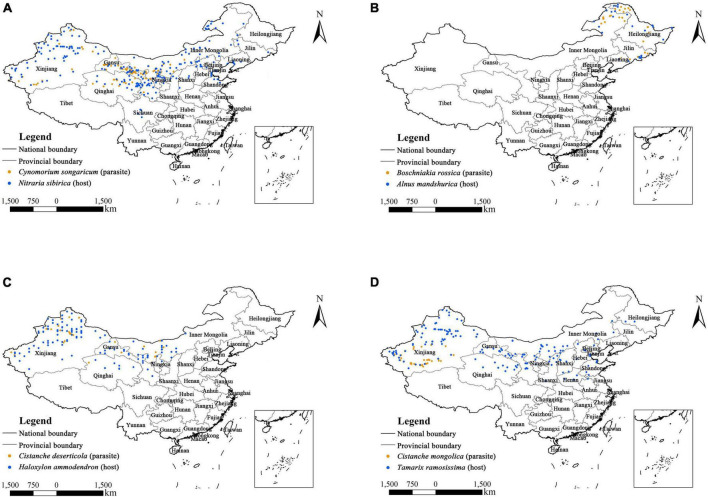
Occurrence records of four holoparasitic plants and their primary hosts in China. **(A)** *Cynomorium songaricum* and *Nitraria sibirica*; **(B)** *Boschniakia rossica* and *Alnus mandshurica*; **(C)** *Cistanche deserticola* and *Haloxylon ammodendron*; **(D)** *Cistanche mongolica* and *Tamarix ramosissima*.

The occurrence data of four parasitic plants and their primary hosts were obtained from three different sources: (1) extensive literature searches [e.g., Web of Science (WOS),^1^ China National Knowledge Infrastructure (CNKI),^2^ and Google Scholar^3^ ], (2) Plant Photo Bank of China (PPBC),^4^ (3) Chinese Virtual Herbarium (CVH)^5^ and National Specimen Information Infrastructure (NSII).^6^ Specifically, the Chinese name, Latin name and common synonyms of each parasitic plant and its host were used as keywords to search for relevant scientific literature (Yang et al., 2012; Chen et al., 2015; Xu et al., 2019; Zhao et al., 2020). We obtained their latitude and longitude information related to exact place names through Google Earth (Google Inc, 2016) and removed duplicated occurrence records. Then, we used the SDMtoolbox (v2.5) in ArcGIS (10.6) to spatially rarefy the occurrence data for each species in this study. Specifically, we established a 1 km × 1 km grid in ArcGIS 10.6 to ensure that there was only one point in each grid. Finally, the geographic coordinate information on the distribution of *Cynomorium songaricum* (121 points), *Boschniakia rossica* (37 points), *Cistanche deserticola* (33 points), *Cistanche mongolica* (46 points), *Nitraria sibirica* (229 points), *Alnus mandshurica* (34 points), *Haloxylon ammodendron* (130 points), and *Tamarix ramosissima* (168 points) was saved in “.csv” format for modeling (Supplementary Table 1). In addition, their occurrence records are shown in Figure 2.

### Climate data

Temperature and precipitation are two key factors affecting the distribution of species (Qin et al., 2020). Thus, 19 bioclimatic variables affecting the distribution of parasitic plants and their primary hosts, which have higher biological relevance and are widely used in ecological modeling (Evangelista et al., 2011; Layola et al., 2022), were selected for this study. Although WorldClim 2.1 version was released in 2017 by Fick and Hijmans (2017), WorldClim 1.4 version^7^ has been widely used for species potential distribution prediction (Jarvie and Svenning, 2018; Ren et al., 2020; Cerasoli et al., 2022). Thus, the 19 bioclimatic variables (Bio1-Bio19, Supplementary Table 2) for the current (1960–1990, Hijmans et al., 2005) and future (2050s and 2070s) climate scenarios in our study were obtained from the WorldClim 1.4 version with a spatial resolution of 30 s (approximately 1 km^2^). For future climate scenarios, WorldClim 1.4 version provides climate data from 19 global climate models (GCMs), but no single climate model is superior in forecasting future climate. Therefore, we followed the method of Chen et al. (2020) who assumed an averaged multi-model ensemble climate forecast for distribution projection in future scenarios. We downloaded the future climate data of three GCMs (Beijing Climate Centre Climate System Model 1.1, BCC_CSM1.1; the Community Climate System Model version 4, CCSM4; the Model for Interdisciplinary Research on Climate, MIROC-ESM) under two representative concentration pathways (RCP2.6 and RCP8.5). They represent the lowest greenhouse gas emission scenario (RCP2.6) and the highest scenario (RCP8.5), respectively (Mackay, 2008; Sun et al., 2020). Then, we calculated the equally-weighted mean values of the three GCMs as a set of future climate data. Climate data in “.tif” format were converted to “.asc” format in ArcGIS 10.6 (Environmental Systems Resource Institute, ESRI) (Asadalla et al., 2021).

Additionally, strongly correlated bioclimatic variables can lead to overfitting of the model. To avoid this, we used Pearson’s correlation coefficient (*r*) method to reduce multicollinearity among the 19 bioclimatic variables. If | *r* | ≤ 0.7, the bioclimatic variable was retained. For the two variables with | *r* | > 0.7, the one with a smaller contribution rate was eliminated (Dormann et al., 2013). The bioclimatic variables included in the MaxEnt model of four holoparasitic plants and their primary hosts were shown in Supplementary Table 3.

### Model simulation and evaluation

The MaxEnt 3.4.1 software (Princeton University, United States), based on the distribution data of species and the environment variables, was used to predict the potential distribution of the four parasitic plants and their primary hosts under the current and future climate scenarios. As a relatively universal model, MaxEnt performs better than other species distribution models (SDMs), especially when the number of distribution records is small (Filz and Schmitt, 2015; Dube et al., 2022). We used the ENMeval package in R 4.2.0 to select the optimal model tuning parameters for each species. We set the regularization multiplier (RM) values ranging from 0.5 to 4 (increments of 0.5) and six feature class (FC) combination (L, H, LQ, LQH, LQHP, LQHPT; L: linear; Q: quadratic; H: hinge; P: product; T: threshold). When delta.AICc value is 0, we consider corresponding RM and FC as the optimal model tuning parameters (Muscarella et al., 2014; Chen et al., 2020). In this study, 75% of the distribution data were set as training data, and the remaining 25% were testing data. To ensure the accuracy of the results, 10000 background points and 15 replications were performed. According to the results of model optimization (Supplementary Table 4), we set RM and FC parameters in MaxEnt models for each species.

Furthermore, we evaluated model performance using the area under curve (AUC) from the receiver operating characteristic (ROC) curve and the true skill statistic (TSS) because their combination can better assess the model performance (Wang et al., 2019; Ren et al., 2020). The AUC, ranging from 0.5 (random prediction) to 1.0 (perfect prediction), is threshold independent (Manel et al., 2001; Chen et al., 2020). Generally, the AUC was classified into five groups: (1) excellent: 0.90–1.00; (2) good: 0.80–0.90; (3) fair: 0.70–0.80; (4) poor: 0.60–0.70; (5) failing: 0.50–0.60 (Phillips and Dudík, 2008; Jalaeian et al., 2018). The TSS value is threshold dependent and calculated as: TSS = Sensitivity + Specificity – 1. It ranges from –1 to 1, where 1 indicates perfect performance, and 0 or less indicates a model performance no better than the random (Allouche et al., 2006; Jiang et al., 2022). MaxEnt produced a prediction China map based on a logistic output format, which shows a continuous habitat suitability index (HSI), ranging from 0 (unsuitable) to 1 (perfectly suitable) (Wang et al., 2019). The output results of “.asc” format were imported into ArcGIS 10.6 for rasterization, maps visualization, and suitability classification of HSI. Habitat suitability was reclassified into four categories: (1) not suitable habitat: 0.00–0.25; (2) low suitable habitat: 0.25–0.50; (3) moderately suitable habitat: 0.50–0.75; (4) highly suitable habitat: 0.75–1.00 (Ren et al., 2020).

### Niche overlap metrics

ENMTools 1.3.1 was used to calculate ecological niche overlap in terms of Schoener’s *D* between parasitic plants and their primary hosts under different climate scenarios. The formula is as follows (Warren et al., 2008, 2010):


$$D\,\left(p_{X},p_{Y}\right)=1-\frac{1}{2}\,\sum_{i}\left|p_{X,i}-p_{Y,i}\right|$$

where *p*_X,i_ (or *p*_Y,i_) represents the normalized suitability scores for a parasitic plant *X* (its host plant *Y*) in grid cell *i*.

The value of Schoener’s *D* ranges from 0 (no similarity) to 1 (identical potential distribution), which describes the degree of similarity of potential distributions by comparing corresponding values per cell of two grids (Broennimann et al., 2012). Generally, the Schoener’s *D* was classified into five classes to facilitate the interpretation of results: (1) very high overlap: 0.80–1.00; (2) high overlap: 0.60–0.80; (3) moderate overlap: 0.40–0.60; (4) low overlap: 0.20–0.40; (5) no or very limited overlap: 0.00–0.20 (Rödder and Engler, 2011; Hyseni and Garrick, 2019).

In addition, a one-way analysis of variance (ANOVA) and the Tamhane test was applied to identify significant differences of niche overlap in terms of Schoener’s *D* among four parasite-host pairs under different climate scenarios. The statistics analysis was performed using SPSS 20 for Windows (SPSS, Inc., Chicago, IL, United States) (Wu, 2019).

## Results

### Model performance and contribution of climatic variables

The mean AUC values of 15 replications of four endangered parasitic plants and their primary hosts were greater than 0.80. Specifically, the AUC values of *Nitraria sibirica* and *Tamarix ramosissima* ranged from 0.8 to 0.9, while the other six species were all greater than 0.9. Similarly, the mean value of TSS for the two species were greater than 0.58 and for the other species were greater than 0.81 (Table 1). Therefore, this indicates that the MaxEnt model performed well in terms of accuracy and reliability under current and future climatic scenarios.

**TABLE 1:** Area under curve and TSS values of four holoparasitic plants and their primary hosts under different climate scenarios in China.

No.	Species	Climate scenarios									
		Current climate		RCP2.6-2050s		RCP8.5-2050s		RCP2.6-2070s		RCP8.5-2070s	
		AUC	TSS	AUC	TSS	AUC	TSS	AUC	TSS	AUC	TSS
1	P: *Cynomorium songaricum*	0.937	0.767	0.936	0.763	0.938	0.755	0.937	0.758	0.937	0.749
	H: *Nitraria sibirica*	0.844	0.597	0.833	0.593	0.824	0.579	0.831	0.583	0.830	0.587
2	P: *Boschniakia rossica*	0.954	0.835	0.949	0.828	0.946	0.806	0.949	0.810	0.949	0.821
	H: *Alnus mandshurica*	0.964	0.865	0.962	0.874	0.959	0.873	0.960	0.869	0.960	0.852
3	P: *Cistanche deserticola*	0.948	0.827	0.953	0.837	0.952	0.825	0.949	0.824	0.952	0.828
	H: *Haloxylon ammodendron*	0.906	0.723	0.900	0.685	0.902	0.681	0.901	0.696	0.902	0.687
4	P: *Cistanche mongolica*	0.980	0.906	0.975	0.878	0.974	0.879	0.975	0.881	0.975	0.868
	H: *Tamarix ramosissima*	0.848	0.586	0.845	0.576	0.844	0.569	0.842	0.583	0.845	0.579

P, parasite; H, host.

Furthermore, we examined the percentage contribution of each climatic variable in MaxEnt model of the eight plants (Supplementary Table 3) by the jackknife method, and selected the value greater than 10.0% as the key climate factor affecting the potential distribution of every species (Table 2). For parasitic *Cynomorium songaricum*, Mean diurnal range (Bio2), Temperature annual range (Bio7), Mean temperature of coldest quarter (Bio11), Precipitation of wettest quarter (Bio16), and Precipitation of driest quarter (Bio17) contributed the maximum, with a total percentage contribution of 96.9%. For its host *Nitraria sibirica*, Min temperature of coldest month (Bio6), Bio7 and Bio17 were the three most important variables, with a total percentage contribution of 79.4%. Bio7 and Bio17 were thus the common key climate factors influencing the distribution of the parasite-host pair. For parasitic *Boschniakia rossica* and its host *Alnus mandshurica*, they shared two common climate factors, namely Temperature seasonality (Bio4), and Precipitation of driest month (Bio14), with total percentage contributions of 84.0 and 59.8%, respectively. For parasitic *Cistanche deserticola*, Bio4, Annual precipitation (Bio12), Precipitation seasonality (Bio15), and Precipitation of warmest quarter (Bio18) were the four most important variables, with a total percentage contribution of 87.0%. For its host *Haloxylon ammodendron*, the first two leading variables were Bio4 and Precipitation of wettest month (Bio13), with a total percentage contribution of 73.0%. In contrast, they shared only one common climate factor (Bio4). For *Cistanche mongolica*, the first three leading factors were Bio11, Bio14, and Bio18, with a total percentage contribution of 98.5%. For its host *Tamarix ramosissima*, the first three factors were Bio4, Bio6, and Bio18, with a total percentage contribution of 80.6%. They also shared only one common climate factor (Bio18).

**TABLE 2:** Key climatic factors influencing habitat distribution of four holoparasitic plants and their primary hosts in China.

No.	Species	Climatic factors	Total percentage contribution (%)
1	P: *Cynomorium songaricum*	Bio2, Bio7, Bio11, Bio16, Bio17	96.9
	H: *Nitraria sibirica*	Bio6, Bio7, Bio17	79.4
2	P: *Boschniakia rossica*	Bio4, Bio10, Bio14	97.2
	H: *Alnus mandshurica*	Bio4, Bio9, Bio14	90.8
3	P: *Cistanche deserticola*	Bio4, Bio12, Bio15, Bio18	87.0
	H: *Haloxylon ammodendron*	Bio4, Bio13	73.0
4	P: *Cistanche mongolica*	Bio11, Bio14, Bio18	98.5
	H: *Tamarix ramosissima*	Bio4, Bio6, Bio18	80.6

P, parasite; H, host.

The climatic variable with percentage contribution > 10.0% was listed as a key climatic factor in Table 2.

### Suitable habitats of four holoparasites and their primary hosts

**FIGURE 3:**
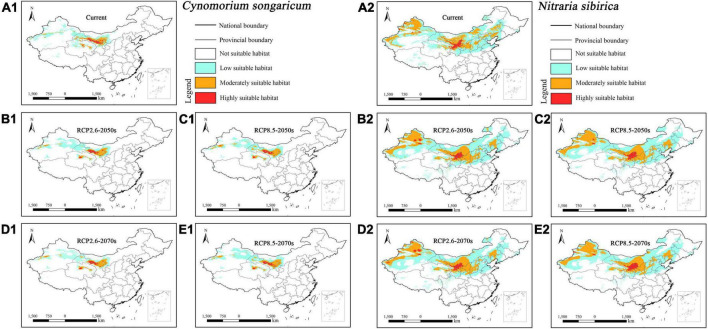
Predicted suitable habitat distributions of *Cynomorium songaricum* **(A1–E1)** and its host *Nitraria sibirica* **(A2–E2)** under different climate scenarios in China.

The suitable habitat (>0.5, moderately and highly suitable habitat) of parasitic *Cynomorium songaricum* concentrated in central Xinjiang, central Qinghai, central Gansu, and southwestern Inner Mongolia under current and future climatic scenarios (Figures 3A1–E1). In contrast, its host *Nitraria sibirica* concentrated in western Xinjiang, most of Inner Mongolia, central Qinghai, central Gansu, central and northern Ningxia, northern Shaanxi, southern Shanxi, and central Hebei (Figures 3A2–E2). Overall, the suitable habitat of host *Nitraria sibirica* was much larger than its parasitic *Cynomorium songaricum*.

**FIGURE 4:**
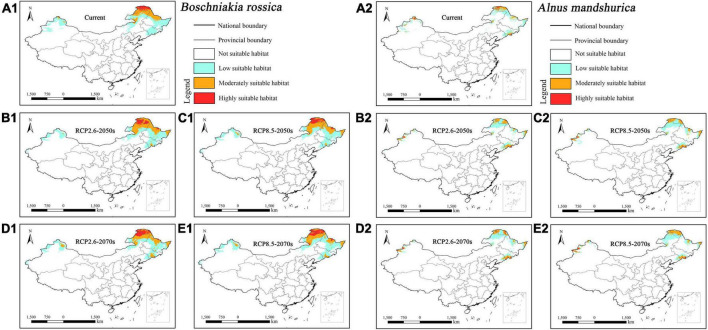
Predicted suitable habitat distributions of *Boschniakia rossica* **(A1–E1)** and its host *Alnus mandshurica* **(A2–E2)** under different climate scenarios in China.

The suitable habitat of parasitic *Boschniakia rossica* concentrated in northern Inner Mongolia, southeastern Jilin, and central and northern Heilongjiang under current and future climatic scenarios (Figures 4A1–E1). Additionally, the parasite would slightly migrate toward southeast of China with the change in climate. In contrast, its host *Alnus mandshurica* concentrated in southeast Jilin and Heilongjiang (Figures 4A2–E2). Overall, the suitable habitat of host *Alnus mandshurica* was smaller than its parasitic *Boschniakia rossica*.

**FIGURE 5:**
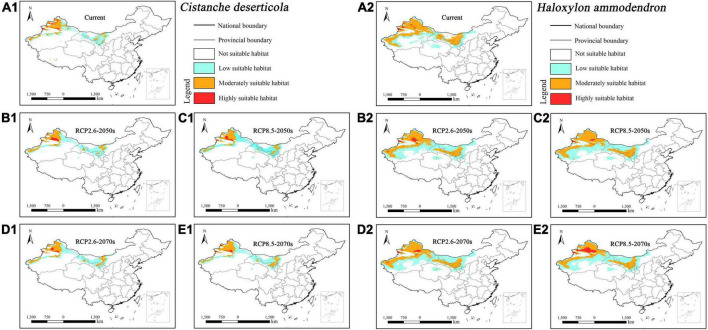
Predicted suitable habitat distributions of *Cistanche deserticola* **(A1–E1)** and its host *Haloxylon ammodendron* **(A2–E2)** under different climate scenarios in China.

The suitable habitat of parasitic *Cistanche deserticola* concentrated in northwestern Xinjiang and central Inner Mongolia under current and future climatic scenarios (Figures 5A1–E1). In contrast, its host *Haloxylon ammodendron* concentrated in northwestern Xinjiang, central and southwestern Inner Mongolia, and central Gansu (Figures 5A2–E2). Overall, the suitable habitat of host *Haloxylon ammodendron* was larger than its parasitic *Cistanche deserticola*.

**FIGURE 6:**
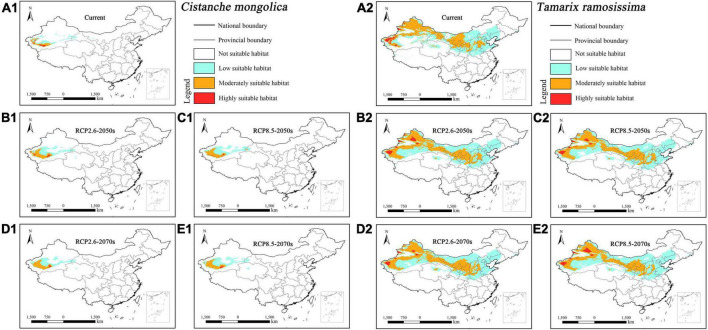
Predicted suitable habitat distributions of *Cistanche mongolica* **(A1–E1)** and its host *Tamarix ramosissima* **(A2–E2)** under different climate scenarios in China.

The suitable habitat of parasitic *Cistanche mongolica* concentrated in southwestern Xinjiang under current and future climatic scenarios (Figures 6A1–E1). In contrast, its host *Tamarix ramosissima* concentrated in most of Xinjiang, central and southwestern Inner Mongolia, central Qinghai, central Gansu, northern and central Ningxia, central Shaanxi, central Shanxi, and southern Hebei (Figures 6A2–E2). Overall, the suitable habitat of host *Tamarix ramosissima* was considerably larger than its parasitic *Cistanche mongolica*.

### Variation in habitat area of four holoparasites and their primary hosts under climate change

Under current climate scenario, the four holoparasites had small suitable habitat areas, ranging from 0.97% (i.e., *Cistanche mongolica*) to 3.77% (i.e., *Boschniakia rossica*) of China’s total area, of which the highly suitable habitat areas accounted for only from 0.19% (i.e., *Cistanche mongolica*) to 0.81% (i.e., *Boschniakia rossica*) (Table 3). Under future climate scenarios, their suitable distribution areas changed to some extent, but with a wide range and different trends. Under RCP2.6-2050s, RCP2.6-2070s, RCP8.5-2050s, and RCP8.5-2070s scenarios, the suitable habitat areas of *Cynomorium songaricum* covered 2.37, 2.42, 2.69, and 2.59%, respectively, indicating that they changed little compared with its current area (2.68%). In contrast, the suitable habitat areas of *Cistanche mongolica* accounted for 1.64, 1.55, 1.61, and 1.62%, respectively; each of them was more than 1.6 times as much as the current area. For these holoparasites, compared to the current, their variation in projected suitable habitats was roughly classified into two categories: growing type (i.e., *Boschniakia rossica* and *Cistanche mongolica*) and fluctuating type (i.e., *Cynomorium songaricum* and *Cistanche deserticola*). For *Boschniakia rossica* and *Cistanche mongolica*, climate change caused their suitable distribution areas to increase in the future. Compared with the current results (3.77 and 0.97%, respectively), the maximum of their suitable habitats appeared in 2050s under RCP8.5 and RCP2.6 scenarios, increasing by 1.14 and 0.67%, respectively. For *Cynomorium songaricum*, its suitable distribution area increased slightly only in 2050s under RCP8.5 scenario, while decreased under the other three scenarios (i.e., RCP2.6-2050s, RCP2.6-2070s, RCP8.5-2070s). For *Cistanche deserticola*, its suitable distribution area increased slightly only in 2070s under RCP8.5 scenario, while decreased under the other three scenarios (i.e., RCP2.6-2050s, RCP8.5-2050s, RCP2.6-2070s). In addition, except for *Boschniakia rossica*, the highly suitable habitats of the other three holoparasites were less than 0.50% in China under future climate scenarios.

**TABLE 3:** Dynamics of changes in distribution area of four holoparasitic plants and their primary hosts under different climate scenarios.

Climate scenarios	Not suitable habitat (%)	Low suitable habitat (%)	Moderately suitable habitat (%)	Highly suitable habitat (%)	Suitable habitat (moderately and highly) (%)
***Cynomorium songaricum*** **(Parasite)**					
Current climate	91.35	5.97	2.13	0.55	2.68
RCP2.6-2050s	91.69	5.95	1.93	0.44	2.37
RCP8.5-2050s	91.71	5.61	2.24	0.45	2.69
RCP2.6-2070s	91.65	5.93	2.01	0.41	2.42
RCP8.5-2070s	90.89	6.51	2.16	0.43	2.59
***Nitraria sibirica*** **(Host)**					
Current climate	65.91	22.28	10.81	1.00	11.81
RCP2.6-2050s	62.21	24.81	11.94	1.03	12.97
RCP8.5-2050s	61.96	24.77	12.30	0.97	13.27
RCP2.6-2070s	62.58	24.69	11.54	1.20	12.74
RCP8.5-2070s	62.78	24.34	11.74	1.15	12.89
***Boschniakia rossica*** **(Parasite)**					
Current climate	88.62	7.62	2.96	0.81	3.77
RCP2.6-2050s	87.99	7.13	3.99	0.89	4.88
RCP8.5-2050s	88.48	6.61	3.96	0.95	4.91
RCP2.6-2070s	88.54	6.73	3.62	1.11	4.73
RCP8.5-2070s	88.52	7.06	3.28	1.14	4.42
***Alnus mandshurica*** **(Host)**					
Current climate	95.05	3.53	1.24	0.18	1.42
RCP2.6-2050s	95.14	3.29	1.47	0.10	1.57
RCP8.5-2050s	94.53	3.71	1.66	0.10	1.76
RCP2.6-2070s	94.83	3.54	1.43	0.20	1.63
RCP8.5-2070s	94.49	3.61	1.73	0.16	1.89
***Cistanche deserticola*** **(Parasite)**					
Current climate	91.01	5.35	3.27	0.38	3.65
RCP2.6-2050s	90.58	5.96	3.04	0.42	3.46
RCP8.5-2050s	90.56	5.97	3.14	0.34	3.48
RCP2.6-2070s	90.35	6.03	3.34	0.28	3.62
RCP8.5-2070s	90.26	6.07	3.40	0.28	3.68
***Haloxylon ammodendron*** **(Host)**					
Current climate	83.11	7.29	9.31	0.29	9.60
RCP2.6-2050s	79.78	11.31	8.41	0.50	8.91
RCP8.5-2050s	79.48	11.42	8.86	0.24	9.10
RCP2.6-2070s	79.65	11.26	8.69	0.40	9.09
RCP8.5-2070s	80.28	11.22	7.41	1.10	8.51
***Cistanche mongolica*** **(Parasite)**					
Current climate	97.33	1.70	0.78	0.19	0.97
RCP2.6-2050s	95.59	2.77	1.51	0.13	1.64
RCP8.5-2050s	95.31	3.08	1.52	0.09	1.61
RCP2.6-2070s	95.67	2.77	1.48	0.07	1.55
RCP8.5-2070s	95.32	3.07	1.53	0.09	1.62
***Tamarix ramosissima*** **(Host)**					
Current climate	68.61	17.96	12.75	0.68	13.43
RCP2.6-2050s	66.09	19.74	13.33	0.83	14.16
RCP8.5-2050s	66.13	20.15	13.09	0.63	13.72
RCP2.6-2070s	65.60	20.11	13.77	0.52	14.29
RCP8.5-2070s	66.17	20.71	12.19	0.93	13.12

In contrast, under current climate scenario, the four host species varied significantly in suitable habitat areas, ranging from 1.42% (i.e., *Alnus mandshurica*) to 13.43% (i.e., *Tamarix ramosissima*). They all had highly restricted suitable habitats of 0.18% (i.e., *Alnus mandshurica*) –1.00% (i.e., *Nitraria sibirica*) (Table 3). Their suitable habitat areas changed slightly in the future, but with distinct trends of variation: growing type (i.e., *Nitraria sibirica* and *Alnus mandshurica*), declining type (i.e., *Haloxylon ammodendron*) and fluctuating type (i.e., *Tamarix ramosissima*). For *Nitraria sibirica*, climate change resulted in expanding its suitable distribution area. The maximum (13.27%) of its suitable habitat appeared in 2050s under RCP8.5 scenario, increasing by 1.46% compared with the current area (11.81%). For *Alnus mandshurica*, its suitable distribution also increased in the future. The maximum (1.89%) of its suitable habitat appeared in 2070s under RCP8.5 scenario, increasing by 0.47% compared with the current area (1.42%). *Haloxylon ammodendron* had the suitable habitat area of 9.60% under the current scenario. Its suitable habitat decreased under future climate scenarios. The minimum (8.51%) appeared in RCP8.5-2070s, decreasing by 1.09%. Compared with the current result (13.43%), the suitable habitat area of *Tamarix ramosissima* fluctuated under future climate scenarios. It decreased by 0.31% in 2070s under RCP8.5 scenario, while increased under the other three future climate scenarios. In addition, except for *Nitraria sibirica* (1.20%, RCP2.6-2070s scenario; 1.15%, RCP8.5-2070s), the highly suitable habitat areas of all the other hosts were no more than 1.10% in China under future climate scenarios.

### Variation in niche overlap between holoparasites and their primary hosts

As shown in Table 4, the niche overlaps between holoparasites and their primary hosts fell into three categories: 0.2–0.4 (low overlap), 0.4–0.6 (moderate overlap), 0.6–0.8 (high overlap). The niche overlaps between *Boschniakia rossica* and *Alnus mandshurica* were greater than 0.6 (high overlap) in all climate scenarios, with the highest overlap of 0.7316 (RCP8.5-2070s scenario). In contrast, the niche overlap between *Cistanche mongolica* and *Tamarix ramosissima* was comparatively low. The minimum (0.2593) appeared under current climate scenario; the maximum (only 0.2908) under RCP2.6-2050s scenario. In addition, the results of ANOVA showed that there was a highly significant difference (*P* < 0.01) among the niche overlaps of the four parasite-host pairs.

**TABLE 4:** Niche overlap in terms of Schoener’s *D* between each holoparasitic plant and its primary host under different climate scenarios.

Climate scenarios	Parasite-Host			
	*C. songaricum-N. sibirica*	*B. rossica-A. mandshurica*	*C. deserticola-H. ammodendron*	*C. mongolica-T. ramosissima*
Current climate	0.5180	0.6673	0.6585	0.2593
RCP2.6-2050s	0.5238	0.6769	0.6684	0.2908
RCP8.5-2050s	0.5080	0.7067	0.6577	0.2853
RCP2.6-2070s	0.5115	0.7208	0.6792	0.2846
RCP8.5-2070s	0.5096	0.7316	0.6746	0.2723
Mean ± SD	0.5142 ± 0.0066*^b^*	0.7007 ± 0.0277*^a^*	0.6677 ± 0.0096*^a^*	0.2785 ± 0.0127*^c^*

*C. songaricum, Cynomorium songaricum; N. sibirica, Nitraria sibirica; B. rossica, Boschniakia rossica; A. mandshurica, Alnus mandshurica; C. deserticola, Cistanche deserticola; H. ammodendron, Haloxylon ammodendron; C. mongolica, Cistanche mongolica; T. ramosissima, Tamarix ramosissima.*

Mean ± SD refers to the average value of Schoener’s *D* of each parasite-host pair under current and four future climate scenarios. Groups identified by different letters are significantly different in the same column (*P* < 0.01).

Compared to the current, the projected niche overlaps among four parasite-host pairs showed distinct variation trend, which can be classified into two categories: growing type (*Boschniakia rossica* vs. *Alnus mandshurica*, and *Cistanche mongolica* vs. *Tamarix ramosissima*), and fluctuating type (*Cynomorium songaricum* vs. *Nitraria sibirica*, and *Cistanche deserticola* vs. *Haloxylon ammodendron*). The niche overlap between *Cynomorium songaricum* and *Nitraria sibirica* was 0.5180 in the current. It then fluctuated under future climate change scenario, with the maximum of 0.5238 (RCP2.6-2050s). Similarly, the niche overlap between *Cistanche deserticola* and *Haloxylon ammodendron* also fluctuated under future climate scenarios. Under current condition, their niche overlap was 0.6585, while it decreased to 0.6577 under RCP8.5-2050s and increased under the other three future scenarios. The maximum (0.6792) appeared in 2070s under RCP2.6 scenario. In contrast, climate change increased the niche overlap between *Boschniakia rossica* and *Alnus mandshurica*, with the maximum of 0.7316 (RCP8.5-2070s scenario). For *Cistanche mongolica* and *Tamarix ramosissima*, the niche overlap between them also increased under future climate scenarios, with the maximum of 0.2908 in 2050s under RCP2.6 scenario.

## Discussion

### Key bioclimatic factors influencing suitable habitats of holoparasitic plants and their hosts

Environmental factors affect the growth, development and distribution of plants (Hamann et al., 2021; Li and Zhang, 2021). Climate is one of the key factors shaping the future distribution of plants (Gomes et al., 2020; Tang et al., 2021). Based on the MaxEnt modeling, our results showed that the key climate factors affecting the four holoparasitic plants were markedly different, which suggested that although these parasites were all endangered perennial herbs and root holoparasites, they responded distinctively to climate change. Moreover, such a difference in response may be related to species characteristics. For example, *Boschniakia rossica* likes to grow in a shady, humid and low-temperature environment (Zhang and Zhang, 2015), while *Cistanche deserticola* in arid or semi-arid desert areas with low annual rainfall and long sunshine duration (Wang et al., 2012).

The growth and distribution of parasitic plants are influenced by environmental factors (i.e., climate, soil, topography), and by biological factors (i.e., competition, mimicry, herbivory) (Heide-Jørgensen, 2008; Zhang et al., 2018; Jiang and Zhang, 2021). Based on ArcGIS-based MaxEnt modeling, we found that among the four parasite-host pairs, *Cynomorium songaricum* and *Nitraria sibirica* had two common climate factors: Bio7 and Bio17, *Boschniakia rossica* and *Alnus mandshurica* also had two ones: Bio4 and Bio14 (Table 2). For *Cistanche deserticola* and *Haloxylon ammodendron*, they had only one common climate factor: Bio4. For *Cistanche mongolica* and *Tamarix ramosissima*, they had only one common climate factor, namely Bio18. Therefore, it seems likely that different parasite-host pairs share to varying degree the common climatic factors influencing their habitat distribution.

### Mismatch in projected distribution between holoparasites and their primary hosts

The results of our species distribution models showed that the suitable habitat areas of the four holoparasitic plants under future climate scenarios were all relatively small, among which the maximum area of highly suitable habitat was only 1.14% (Table 3). Based on the variation trends of their projected suitable habitats, we identified two categories: growing type (*Boschniakia rossica* and *Cistanche mongolica*), and fluctuating type (*Cynomorium songaricum* and *Cistanche deserticola*). This finding is different from the results reported by Shao et al. (2022). They forecasted the suitable distribution of *Cistanche deserticola* would expand under future climatic scenarios in northwest China based on the MaxEnt model. However, Mkala et al. (2022) inferred that the potential distribution of two holoparasitic species of *Hydnora* would decrease in Africa in the future. Therefore, we demonstrate that different species of holoparasites may response differently to future climate change.

Our results also showed that the four hosts had different suitable habitat areas under future climate scenarios. On the whole, their distribution shift presented three categories: growing type (*Nitraria sibirica* and *Alnus mandshurica*), declining type (*Haloxylon ammodendron*) and fluctuating type (*Tamarix ramosissima*). This indicates that host plants may respond differently to future climate change. Although *Nitraria sibirica* and *Alnus mandshurica* were of growth types, they increased slightly and fluctuated considerably in terms of highly suitable habitat area under four future climate scenarios (Table 3). *Haloxylon ammodendron*, a small xerophytic tree, grows in sandy deserts with strong tolerance to drought, wind-erosion, and salt-alkali (Wu et al., 2003; He et al., 2021). Accordingly, it is usually a dominant species of sandy vegetation in northern China. The decrease of its suitable habitat under future climate scenarios may lead to the degradation of its community habitat, which will be detrimental to the growth and distribution of *Haloxylon ammodendron*. *Tamarix ramosissima* is a small tree, and mainly distributed in northern China including Xinjiang, Inner Mongolia, Ningxia, and Gansu (Figures 6A2–E2). Our MaxEnt modeling showed that its suitable habitat area decreased under RCP8.5-2070s scenario, thus resulting in its population decline while its suitable habitat area increased under the other three future scenarios, resulting in its population growing. This tree is taken as a primary host of *Cistanche mongolica* (Wu et al., 2007). Such a rapid fluctuation in host population quantity may inevitably affect the corresponding parasite’s growth and distribution in the future.

In addition, we noticed that the variation trends of suitable habitats of these parasites were not consistent with counterparts of their primary hosts under future climate scenarios. For example, *Cynomorium songaricum* was of fluctuating type in the aspect of future distribution while its host *Nitraria sibirica* was of growing type. Therefore, there exists a spatial mismatch in the projected suitable habitats between parasites and their primary hosts, and furthermore this will have an adverse impact on distribution of parasites and their hosts in the future. The selected parasitic plants in this study are all obligate, and they exhibit high degree of host dependence. For example, *Cistanche deserticola* mainly parasitizes the root of *Haloxylon ammodendron* (Liu et al., 2019). It is difficult for a parasitic plant to develop its novel host specificity within several decades, especially for a holoparasite. Therefore, such a spatial mismatch exacerbates the impacts of global warming on suitable habitats of endangered parasites, and meanwhile it poses a great challenge to the host specificity of these parasites.

### Alteration of ecological niche overlap between holoparasites and their primary hosts in the future

In terms of Schoener’s *D* value, the niche overlaps of the four parasite-host pairs had the mean value of 0.2785–0.7007 (Table 4), which indicated that there were significant differences in the niche overlaps among different parasite-host pairs (*P* < 0.01). The niche overlap between *Boschniakia rossica* and *Alnus mandshurica* was the highest (0.7007 ± 0.0277, Mean ± SD), followed by *Cistanche deserticola* and *Haloxylon ammodendron* (0.6677 ± 0.0096) and *Cynomorium songaricum* and *Nitraria sibirica* (0.5142 ± 0.0066). The lowest niche overlap was found between *Cistanche mongolica* and *Tamarix ramosissima* (0.2785 ± 0.0127).

Based on variation trends of niche overlaps among the four parasite-host pairs, we identified two categories: growing type (*Boschniakia rossica* vs. *Alnus mandshurica* and *Cistanche mongolica* vs. *Tamarix ramosissima*), and fluctuating type (the other two pairs). Firstly, the niche overlap between *Boschniakia rossica* and *Alnus mandshurica* is consistent with their suitable habitat shift (see section “Mismatch in projected distribution between holoparasites and their primary hosts”). However, there is a notable difference in highly suitable habitat that the former belongs to growing type while the latter to fluctuating one. Such a spatial mismatch in the highly suitable habitat will inevitably decrease the access to nutrients and water for parasites, and thus influencing their growth and distribution. Secondly, the niche overlap between *Cistanche mongolica* and *Tamarix ramosissima* will increase under future climate scenarios (Table 3). However, *Cistanche mongolica* is of growing type while its host is of fluctuating one in terms of suitable habitat shift. Similarly, future climate change has adverse effect on the growth of this holoparasite. Thirdly, the niche overlaps of the other two pairs are of fluctuating type. However, *Cynomorium songaricum* and *Cistanche deserticola* are of fluctuating type while their hosts *Nitraria sibirica* and *Haloxylon ammodendron* are of growing and declining types respectively in terms of suitable habitat shift. Consequently, future climate change may be not conducive to the growth of the two endangered holoparasites. Therefore, although the four parasite-host pairs respond distinctively to climate change, our studies demonstrate that climate change has disadvantageous effect on the growth of the four endangered holoparasites.

This study is the first to predict the habitat distribution of holoparasites and their hosts through niche overlap. Previous studies predicted the distribution mainly by comparing the migration of the geometric centers of suitable areas of a parasite and its host or by regarding the parasite and its host as a single species (Liu et al., 2019; He et al., 2021). We applied the approach of niche overlap (in terms of Schoener’s *D*) (Warren et al., 2008), which is used to compare the distribution similarity between two species, to compare the variation of niche of parasites and their hosts under current and future climate scenarios. Due to simple and convenient manipulation, this approach has been attempted to successfully apply in distribution prediction of organisms at different trophic levels or different species from the same genus in recent years (Qin et al., 2020; Jiang et al., 2022). Our results demonstrate that this method can be applicable to the geographical distribution of parasites. Therefore, our study can provide an important reference for future research on the comparison of suitable habitats for parasites and hosts through niche overlap, especially for obligate parasites and their hosts.

We identified two types of niche overlap change for the four parasite-host pairs in the current study, whereas it remains unknown about which pattern is the major type under future climate scenarios for parasites-hosts. We think that this can be solved by increasing the number of parasite-host pairs in the future.

### Implications for endangered holoparasites’ conservation and management

Currently, the protection of the four endangered holoparasites is facing serious challenges. Firstly, the MaxEnt prediction in this study showed that the suitable habitat areas of the four holoparasites are extremely small (Table 3). Secondly, all of them are distributed in an undeveloped region of northern China, with relatively vulnerable habitats. For example, *Cistanche deserticola* mainly grows in arid or semi-arid desert areas with low annual rainfall (Wang et al., 2012). Thirdly, all these holoparasites have important utilization value, and can be used as the traditional Chinese medicinal materials and nutritional tonics (Wu et al., 2007; Ren et al., 2018). For example, *Cynomorium songaricum*, mainly in desert areas of northern China, has the function of improving male fertility, treating intestinal ailments and enhancing immunity (Yang et al., 2010; Ren et al., 2018), so it is called “Ginseng in desert”. The four endangered holoparasites are in danger because of illegal harvesting, weak conservation awareness, and negligence in conservation management. This has caused their populations to decrease continuously in the last few decades. As a result, all these four holoparasites have been on the *List of National Key Protected Wild Plants* since 2021 (State Forestry and Grassland Administration and the Ministry of Agriculture and Rural Affairs, P. R. China [SFGA], 2021). In addition, all the four endangered holoparasites have strong host dependence. If the hosts’ habitats decrease or deteriorate, this may give rise to negative effect on the survival and development of their corresponding parasites.

In the future, these four endangered holoparasites will be also confronted with some severe problems resulting from global warming. Firstly, although the suitable habitats of *Boschniakia rossica* and *Cistanche mongolica* showed an increasing trend in the future, their highly suitable habitats were all less than 1.15%. At the same time, the suitable habitats of *Cistanche deserticola* and *Cynomorium songaricum* were of fluctuating type. This indicated that future climate change may have an adverse impact on the four holoparasites in suitable distribution areas. Secondly, these holoparasites and their primary hosts respond differently to climate change, which results in an apparent spatial mismatch between them in light of their suitable habitats. Thirdly, niche overlap analysis also indicated that future climate change will be detrimental to the growth of these four holoparasitic plants. Therefore, climate change may put these holoparasites at the risk of a reduction in suitable distribution areas and obligate host availability.

Hence, we propose the following suggestions for conservation of endangered parasitic plants. Firstly, it is necessary to conserve these threatened holoparasites according to their projected distribution, and meanwhile to pay special attention to their distribution shift in suitable habitats under future climate change. Take *Cistanche mongolica* as an example, this species will expand its suitable habitat eastward in Xinjiang, China under future scenarios. Moreover, it will also occur in some areas of southern Gansu Province (Figures 6A1–E1). Secondly, it is also necessary to strengthen the conservation of host plants and their suitable habitats, and to improve the living environment of the primary hosts. For example, *Haloxylon ammodendron*, a primary host of *Cistanche deserticola* (Liu et al., 2019), is one of major tree species for afforestation in desert areas of northern China. Therefore, increased investment in the management of haloxylon forest will contribute to the growth and distribution of this parasitic plant, and to the amelioration of local ecological environment. The last but not least, it is imperative to promote conservation awareness in endangered parasites, and to reasonably use plant resources in light of the close link between parasites and hosts. For instance, the niche overlap score between *Cistanche mongolica* and *Tamarix ramosissima* was the lowest among the four parasite-host pairs under the current and future scenarios (Table 4). Therefore, people should give a prior consideration to this parasite when taking effective measures, which include strengthening its own protection in southwestern Xinjiang, and properly transplanting its host *Tamarix ramosissima* in forest practice.

## Conclusion

We selected four endangered holoparasites and their primary hosts in northern China, and predicted their potential distribution areas using MaxEnt modeling. Our results indicated that there was a pronounced spatial mismatch in projected suitable habitats between parasites and their primary hosts. We also found that climate change has disadvantageous effect on their growth and distribution based on the niche overlap between these holoparasites and their hosts. In addition, this study is the first to predict the habitat distribution of parasitic plants and their hosts through niche overlap approach. In summary, our findings demonstrate that climate factors restricting parasites and hosts’ distributions, niche overlaps between them, together with parasitic species identity may jointly influence the suitable habitats of parasitic plants. Therefore, it is necessary to take into account the threatened holoparasites themselves in conjunction with their suitable habitats, and meanwhile the parasite-host association when making conservation planning in the future.

## Data availability statement

The original contributions presented in the study are included in the article/Supplementary material, further inquiries can be directed to the corresponding author/s.

## Author contributions

XL: analysis and interpretation of data, and writing-original draft. RJ: acquisition and analysis of data, and writing-original draft. GZ: conceiving the study and leading the writing. All authors contributed to the article and approved the submitted version.

## Footnotes

1
https://www.webofscience.com/

2
https://kns.cnki.net/

3
https://scholar.google.com/

4
http://ppbc.iplant.cn

5
http://www.cvh.ac.cn

6
http://www.nsii.org.cn

7
http://www.worldclim.org
