# Supplementary material for: Predicting the potential distribution of four endangered holoparasites and their primary hosts in China under climate change
Source: Front Plant Sci. 2022 Aug 3;13:942448. doi: 10.3389/fpls.2022.942448 (PMC9384867; doi:10.3389/fpls.2022.942448)
Supplement: Supplementary file 1 [file Table_1.DOCX]

**Supplementary Table 1**

Occurrence records of four holoparasitic plants and their primary hosts in China.

| **No. of species** | **Parasite/Host** | **Species** | **Longitude(°)** | **Latitude(°)** |
| --- | --- | --- | --- | --- |
| 1 | parasite | *Cynomorium songaricum* | 75.26 | 39.73 |
| 1 | parasite | *Cynomorium songaricum* | 76.74 | 37.01 |
| 1 | parasite | *Cynomorium songaricum* | 77.54 | 37.95 |
| 1 | parasite | *Cynomorium songaricum* | 79.92 | 36.31 |
| 1 | parasite | *Cynomorium songaricum* | 80.42 | 36.29 |
| 1 | parasite | *Cynomorium songaricum* | 80.76 | 36.54 |
| 1 | parasite | *Cynomorium songaricum* | 81.70 | 37.13 |
| 1 | parasite | *Cynomorium songaricum* | 82.99 | 41.65 |
| 1 | parasite | *Cynomorium songaricum* | 85.28 | 44.88 |
| 1 | parasite | *Cynomorium songaricum* | 87.48 | 43.97 |
| 1 | parasite | *Cynomorium songaricum* | 87.52 | 44.34 |
| 1 | parasite | *Cynomorium songaricum* | 87.58 | 47.67 |
| 1 | parasite | *Cynomorium songaricum* | 87.99 | 39.52 |
| 1 | parasite | *Cynomorium songaricum* | 88.30 | 47.81 |
| 1 | parasite | *Cynomorium songaricum* | 90.05 | 44.21 |
| 1 | parasite | *Cynomorium songaricum* | 90.28 | 43.81 |
| 1 | parasite | *Cynomorium songaricum* | 92.36 | 43.26 |
| 1 | parasite | *Cynomorium songaricum* | 92.37 | 36.48 |
| 1 | parasite | *Cynomorium songaricum* | 94.63 | 35.90 |
| 1 | parasite | *Cynomorium songaricum* | 94.66 | 40.46 |
| 1 | parasite | *Cynomorium songaricum* | 94.85 | 40.39 |
| 1 | parasite | *Cynomorium songaricum* | 94.87 | 40.50 |
| 1 | parasite | *Cynomorium songaricum* | 94.93 | 36.42 |
| 1 | parasite | *Cynomorium songaricum* | 95.00 | 40.55 |
| 1 | parasite | *Cynomorium songaricum* | 95.18 | 39.44 |
| 1 | parasite | *Cynomorium songaricum* | 95.40 | 40.45 |
| 1 | parasite | *Cynomorium songaricum* | 95.57 | 40.47 |
| 1 | parasite | *Cynomorium songaricum* | 95.58 | 40.21 |
| 1 | parasite | *Cynomorium songaricum* | 95.78 | 40.55 |
| 1 | parasite | *Cynomorium songaricum* | 96.21 | 40.26 |
| 1 | parasite | *Cynomorium songaricum* | 96.45 | 36.39 |
| 1 | parasite | *Cynomorium songaricum* | 96.78 | 37.32 |
| 1 | parasite | *Cynomorium songaricum* | 96.90 | 37.32 |
| 1 | parasite | *Cynomorium songaricum* | 97.13 | 41.91 |
| 1 | parasite | *Cynomorium songaricum* | 97.33 | 37.38 |
| 1 | parasite | *Cynomorium songaricum* | 97.47 | 37.22 |
| 1 | parasite | *Cynomorium songaricum* | 97.54 | 37.20 |
| 1 | parasite | *Cynomorium songaricum* | 97.75 | 40.29 |
| 1 | parasite | *Cynomorium songaricum* | 97.93 | 36.01 |
| 1 | parasite | *Cynomorium songaricum* | 98.20 | 39.66 |
| 1 | parasite | *Cynomorium songaricum* | 98.32 | 39.65 |
| 1 | parasite | *Cynomorium songaricum* | 98.32 | 39.98 |
| 1 | parasite | *Cynomorium songaricum* | 98.68 | 39.57 |
| 1 | parasite | *Cynomorium songaricum* | 98.94 | 36.77 |
| 1 | parasite | *Cynomorium songaricum* | 99.00 | 36.81 |
| 1 | parasite | *Cynomorium songaricum* | 99.26 | 39.53 |
| 1 | parasite | *Cynomorium songaricum* | 99.33 | 36.56 |
| 1 | parasite | *Cynomorium songaricum* | 99.42 | 39.50 |
| 1 | parasite | *Cynomorium songaricum* | 99.50 | 39.38 |
| 1 | parasite | *Cynomorium songaricum* | 99.62 | 39.67 |
| 1 | parasite | *Cynomorium songaricum* | 99.75 | 38.93 |
| 1 | parasite | *Cynomorium songaricum* | 99.77 | 38.95 |
| 1 | parasite | *Cynomorium songaricum* | 99.93 | 38.93 |
| 1 | parasite | *Cynomorium songaricum* | 99.93 | 38.96 |
| 1 | parasite | *Cynomorium songaricum* | 100.10 | 39.05 |
| 1 | parasite | *Cynomorium songaricum* | 100.15 | 39.33 |
| 1 | parasite | *Cynomorium songaricum* | 100.18 | 40.02 |
| 1 | parasite | *Cynomorium songaricum* | 100.20 | 38.82 |
| 1 | parasite | *Cynomorium songaricum* | 100.40 | 38.80 |
| 1 | parasite | *Cynomorium songaricum* | 100.45 | 39.38 |
| 1 | parasite | *Cynomorium songaricum* | 100.45 | 39.05 |
| 1 | parasite | *Cynomorium songaricum* | 100.48 | 39.03 |
| 1 | parasite | *Cynomorium songaricum* | 100.50 | 38.73 |
| 1 | parasite | *Cynomorium songaricum* | 100.50 | 39.05 |
| 1 | parasite | *Cynomorium songaricum* | 100.51 | 39.06 |
| 1 | parasite | *Cynomorium songaricum* | 100.55 | 39.73 |
| 1 | parasite | *Cynomorium songaricum* | 100.57 | 38.97 |
| 1 | parasite | *Cynomorium songaricum* | 100.70 | 38.78 |
| 1 | parasite | *Cynomorium songaricum* | 100.71 | 36.76 |
| 1 | parasite | *Cynomorium songaricum* | 100.72 | 38.64 |
| 1 | parasite | *Cynomorium songaricum* | 100.82 | 39.17 |
| 1 | parasite | *Cynomorium songaricum* | 100.84 | 38.86 |
| 1 | parasite | *Cynomorium songaricum* | 100.85 | 39.20 |
| 1 | parasite | *Cynomorium songaricum* | 101.08 | 41.96 |
| 1 | parasite | *Cynomorium songaricum* | 101.12 | 38.88 |
| 1 | parasite | *Cynomorium songaricum* | 101.19 | 38.98 |
| 1 | parasite | *Cynomorium songaricum* | 101.25 | 39.87 |
| 1 | parasite | *Cynomorium songaricum* | 101.30 | 37.60 |
| 1 | parasite | *Cynomorium songaricum* | 101.32 | 39.07 |
| 1 | parasite | *Cynomorium songaricum* | 101.45 | 38.72 |
| 1 | parasite | *Cynomorium songaricum* | 101.60 | 39.33 |
| 1 | parasite | *Cynomorium songaricum* | 101.96 | 39.33 |
| 1 | parasite | *Cynomorium songaricum* | 102.52 | 38.62 |
| 1 | parasite | *Cynomorium songaricum* | 102.92 | 38.43 |
| 1 | parasite | *Cynomorium songaricum* | 103.04 | 38.66 |
| 1 | parasite | *Cynomorium songaricum* | 103.07 | 37.62 |
| 1 | parasite | *Cynomorium songaricum* | 103.10 | 38.57 |
| 1 | parasite | *Cynomorium songaricum* | 103.12 | 34.66 |
| 1 | parasite | *Cynomorium songaricum* | 103.25 | 38.77 |
| 1 | parasite | *Cynomorium songaricum* | 103.34 | 38.94 |
| 1 | parasite | *Cynomorium songaricum* | 103.35 | 39.00 |
| 1 | parasite | *Cynomorium songaricum* | 103.39 | 38.85 |
| 1 | parasite | *Cynomorium songaricum* | 103.53 | 38.88 |
| 1 | parasite | *Cynomorium songaricum* | 103.53 | 39.02 |
| 1 | parasite | *Cynomorium songaricum* | 103.56 | 38.80 |
| 1 | parasite | *Cynomorium songaricum* | 103.60 | 37.59 |
| 1 | parasite | *Cynomorium songaricum* | 103.68 | 38.72 |
| 1 | parasite | *Cynomorium songaricum* | 104.06 | 40.18 |
| 1 | parasite | *Cynomorium songaricum* | 104.15 | 40.17 |
| 1 | parasite | *Cynomorium songaricum* | 104.49 | 37.51 |
| 1 | parasite | *Cynomorium songaricum* | 104.94 | 37.61 |
| 1 | parasite | *Cynomorium songaricum* | 105.01 | 37.49 |
| 1 | parasite | *Cynomorium songaricum* | 105.02 | 37.62 |
| 1 | parasite | *Cynomorium songaricum* | 105.15 | 39.10 |
| 1 | parasite | *Cynomorium songaricum* | 105.39 | 39.23 |
| 1 | parasite | *Cynomorium songaricum* | 105.62 | 39.58 |
| 1 | parasite | *Cynomorium songaricum* | 105.68 | 39.75 |
| 1 | parasite | *Cynomorium songaricum* | 105.83 | 39.67 |
| 1 | parasite | *Cynomorium songaricum* | 106.04 | 38.61 |
| 1 | parasite | *Cynomorium songaricum* | 106.17 | 39.22 |
| 1 | parasite | *Cynomorium songaricum* | 106.72 | 39.47 |
| 1 | parasite | *Cynomorium songaricum* | 106.75 | 39.44 |
| 1 | parasite | *Cynomorium songaricum* | 106.75 | 38.83 |
| 1 | parasite | *Cynomorium songaricum* | 107.00 | 40.24 |
| 1 | parasite | *Cynomorium songaricum* | 107.30 | 40.59 |
| 1 | parasite | *Cynomorium songaricum* | 107.60 | 37.76 |
| 1 | parasite | *Cynomorium songaricum* | 108.72 | 40.50 |
| 1 | parasite | *Cynomorium songaricum* | 109.40 | 39.05 |
| 1 | parasite | *Cynomorium songaricum* | 111.17 | 42.56 |
| 1 | parasite | *Cynomorium songaricum* | 111.71 | 41.54 |
| 1 | parasite | *Cynomorium songaricum* | 112.70 | 42.73 |
| 1 | host | *Nitraria sibirica* | 75.20 | 37.80 |
| 1 | host | *Nitraria sibirica* | 75.30 | 39.70 |
| 1 | host | *Nitraria sibirica* | 76.00 | 39.20 |
| 1 | host | *Nitraria sibirica* | 76.00 | 39.50 |
| 1 | host | *Nitraria sibirica* | 76.20 | 39.70 |
| 1 | host | *Nitraria sibirica* | 77.30 | 38.40 |
| 1 | host | *Nitraria sibirica* | 77.40 | 37.90 |
| 1 | host | *Nitraria sibirica* | 78.50 | 40.90 |
| 1 | host | *Nitraria sibirica* | 78.60 | 39.80 |
| 1 | host | *Nitraria sibirica* | 79.30 | 36.20 |
| 1 | host | *Nitraria sibirica* | 80.30 | 41.20 |
| 1 | host | *Nitraria sibirica* | 80.90 | 44.10 |
| 1 | host | *Nitraria sibirica* | 81.00 | 45.00 |
| 1 | host | *Nitraria sibirica* | 81.10 | 43.20 |
| 1 | host | *Nitraria sibirica* | 81.50 | 44.00 |
| 1 | host | *Nitraria sibirica* | 81.50 | 42.81 |
| 1 | host | *Nitraria sibirica* | 81.70 | 36.90 |
| 1 | host | *Nitraria sibirica* | 82.10 | 44.90 |
| 1 | host | *Nitraria sibirica* | 82.70 | 37.10 |
| 1 | host | *Nitraria sibirica* | 82.90 | 44.60 |
| 1 | host | *Nitraria sibirica* | 83.00 | 41.70 |
| 1 | host | *Nitraria sibirica* | 83.00 | 46.80 |
| 1 | host | *Nitraria sibirica* | 83.30 | 43.40 |
| 1 | host | *Nitraria sibirica* | 83.60 | 45.90 |
| 1 | host | *Nitraria sibirica* | 83.60 | 46.50 |
| 1 | host | *Nitraria sibirica* | 84.08 | 43.84 |
| 1 | host | *Nitraria sibirica* | 84.30 | 41.80 |
| 1 | host | *Nitraria sibirica* | 84.70 | 44.40 |
| 1 | host | *Nitraria sibirica* | 84.90 | 45.60 |
| 1 | host | *Nitraria sibirica* | 84.90 | 44.40 |
| 1 | host | *Nitraria sibirica* | 85.50 | 38.20 |
| 1 | host | *Nitraria sibirica* | 85.60 | 44.30 |
| 1 | host | *Nitraria sibirica* | 85.70 | 46.80 |
| 1 | host | *Nitraria sibirica* | 86.00 | 44.30 |
| 1 | host | *Nitraria sibirica* | 86.20 | 41.90 |
| 1 | host | *Nitraria sibirica* | 86.30 | 41.30 |
| 1 | host | *Nitraria sibirica* | 86.30 | 47.50 |
| 1 | host | *Nitraria sibirica* | 86.30 | 44.30 |
| 1 | host | *Nitraria sibirica* | 86.40 | 42.30 |
| 1 | host | *Nitraria sibirica* | 86.60 | 42.10 |
| 1 | host | *Nitraria sibirica* | 86.90 | 42.30 |
| 1 | host | *Nitraria sibirica* | 86.90 | 44.20 |
| 1 | host | *Nitraria sibirica* | 87.30 | 48.30 |
| 1 | host | *Nitraria sibirica* | 87.40 | 43.90 |
| 1 | host | *Nitraria sibirica* | 87.50 | 44.20 |
| 1 | host | *Nitraria sibirica* | 87.80 | 47.90 |
| 1 | host | *Nitraria sibirica* | 87.84 | 44.32 |
| 1 | host | *Nitraria sibirica* | 87.86 | 44.30 |
| 1 | host | *Nitraria sibirica* | 87.93 | 44.28 |
| 1 | host | *Nitraria sibirica* | 88.00 | 44.00 |
| 1 | host | *Nitraria sibirica* | 88.00 | 46.60 |
| 1 | host | *Nitraria sibirica* | 88.20 | 39.00 |
| 1 | host | *Nitraria sibirica* | 88.30 | 43.40 |
| 1 | host | *Nitraria sibirica* | 88.70 | 42.80 |
| 1 | host | *Nitraria sibirica* | 89.20 | 44.00 |
| 1 | host | *Nitraria sibirica* | 89.20 | 42.90 |
| 1 | host | *Nitraria sibirica* | 89.20 | 42.89 |
| 1 | host | *Nitraria sibirica* | 89.50 | 46.50 |
| 1 | host | *Nitraria sibirica* | 89.60 | 44.00 |
| 1 | host | *Nitraria sibirica* | 93.00 | 44.20 |
| 1 | host | *Nitraria sibirica* | 93.20 | 36.93 |
| 1 | host | *Nitraria sibirica* | 93.30 | 38.10 |
| 1 | host | *Nitraria sibirica* | 93.50 | 42.80 |
| 1 | host | *Nitraria sibirica* | 94.30 | 39.60 |
| 1 | host | *Nitraria sibirica* | 94.70 | 43.30 |
| 1 | host | *Nitraria sibirica* | 94.90 | 39.50 |
| 1 | host | *Nitraria sibirica* | 94.90 | 36.40 |
| 1 | host | *Nitraria sibirica* | 95.58 | 40.21 |
| 1 | host | *Nitraria sibirica* | 95.80 | 40.50 |
| 1 | host | *Nitraria sibirica* | 97.10 | 40.30 |
| 1 | host | *Nitraria sibirica* | 97.40 | 37.40 |
| 1 | host | *Nitraria sibirica* | 98.10 | 36.30 |
| 1 | host | *Nitraria sibirica* | 98.30 | 39.80 |
| 1 | host | *Nitraria sibirica* | 98.50 | 36.90 |
| 1 | host | *Nitraria sibirica* | 98.50 | 39.70 |
| 1 | host | *Nitraria sibirica* | 98.90 | 40.00 |
| 1 | host | *Nitraria sibirica* | 99.00 | 37.30 |
| 1 | host | *Nitraria sibirica* | 99.08 | 36.99 |
| 1 | host | *Nitraria sibirica* | 99.60 | 35.60 |
| 1 | host | *Nitraria sibirica* | 99.60 | 38.80 |
| 1 | host | *Nitraria sibirica* | 99.75 | 38.93 |
| 1 | host | *Nitraria sibirica* | 99.77 | 38.95 |
| 1 | host | *Nitraria sibirica* | 99.93 | 38.93 |
| 1 | host | *Nitraria sibirica* | 100.00 | 36.40 |
| 1 | host | *Nitraria sibirica* | 100.05 | 38.10 |
| 1 | host | *Nitraria sibirica* | 100.18 | 40.02 |
| 1 | host | *Nitraria sibirica* | 100.20 | 37.30 |
| 1 | host | *Nitraria sibirica* | 100.20 | 39.20 |
| 1 | host | *Nitraria sibirica* | 100.50 | 38.90 |
| 1 | host | *Nitraria sibirica* | 100.55 | 39.73 |
| 1 | host | *Nitraria sibirica* | 100.57 | 38.97 |
| 1 | host | *Nitraria sibirica* | 100.60 | 35.20 |
| 1 | host | *Nitraria sibirica* | 100.85 | 39.20 |
| 1 | host | *Nitraria sibirica* | 101.10 | 42.00 |
| 1 | host | *Nitraria sibirica* | 101.50 | 35.90 |
| 1 | host | *Nitraria sibirica* | 101.60 | 39.33 |
| 1 | host | *Nitraria sibirica* | 101.60 | 36.50 |
| 1 | host | *Nitraria sibirica* | 101.70 | 39.20 |
| 1 | host | *Nitraria sibirica* | 101.71 | 36.10 |
| 1 | host | *Nitraria sibirica* | 101.80 | 36.60 |
| 1 | host | *Nitraria sibirica* | 101.80 | 35.90 |
| 1 | host | *Nitraria sibirica* | 101.86 | 36.10 |
| 1 | host | *Nitraria sibirica* | 101.90 | 36.10 |
| 1 | host | *Nitraria sibirica* | 101.96 | 39.33 |
| 1 | host | *Nitraria sibirica* | 101.98 | 30.10 |
| 1 | host | *Nitraria sibirica* | 102.00 | 38.30 |
| 1 | host | *Nitraria sibirica* | 102.00 | 36.40 |
| 1 | host | *Nitraria sibirica* | 102.20 | 36.10 |
| 1 | host | *Nitraria sibirica* | 102.40 | 31.00 |
| 1 | host | *Nitraria sibirica* | 102.40 | 36.50 |
| 1 | host | *Nitraria sibirica* | 102.50 | 35.70 |
| 1 | host | *Nitraria sibirica* | 102.50 | 34.60 |
| 1 | host | *Nitraria sibirica* | 102.60 | 30.90 |
| 1 | host | *Nitraria sibirica* | 102.60 | 37.90 |
| 1 | host | *Nitraria sibirica* | 102.70 | 29.40 |
| 1 | host | *Nitraria sibirica* | 102.74 | 30.10 |
| 1 | host | *Nitraria sibirica* | 102.80 | 36.30 |
| 1 | host | *Nitraria sibirica* | 102.85 | 36.10 |
| 1 | host | *Nitraria sibirica* | 102.90 | 37.50 |
| 1 | host | *Nitraria sibirica* | 103.10 | 38.60 |
| 1 | host | *Nitraria sibirica* | 103.20 | 34.10 |
| 1 | host | *Nitraria sibirica* | 103.30 | 36.70 |
| 1 | host | *Nitraria sibirica* | 103.34 | 38.94 |
| 1 | host | *Nitraria sibirica* | 103.80 | 36.10 |
| 1 | host | *Nitraria sibirica* | 104.00 | 36.30 |
| 1 | host | *Nitraria sibirica* | 104.00 | 34.40 |
| 1 | host | *Nitraria sibirica* | 104.06 | 40.18 |
| 1 | host | *Nitraria sibirica* | 104.10 | 37.20 |
| 1 | host | *Nitraria sibirica* | 104.20 | 36.50 |
| 1 | host | *Nitraria sibirica* | 104.32 | 36.10 |
| 1 | host | *Nitraria sibirica* | 104.70 | 36.60 |
| 1 | host | *Nitraria sibirica* | 104.90 | 33.40 |
| 1 | host | *Nitraria sibirica* | 105.02 | 37.62 |
| 1 | host | *Nitraria sibirica* | 105.10 | 35.70 |
| 1 | host | *Nitraria sibirica* | 105.20 | 37.50 |
| 1 | host | *Nitraria sibirica* | 105.62 | 39.58 |
| 1 | host | *Nitraria sibirica* | 105.70 | 38.90 |
| 1 | host | *Nitraria sibirica* | 105.70 | 36.70 |
| 1 | host | *Nitraria sibirica* | 105.70 | 34.60 |
| 1 | host | *Nitraria sibirica* | 105.83 | 39.67 |
| 1 | host | *Nitraria sibirica* | 105.90 | 37.00 |
| 1 | host | *Nitraria sibirica* | 106.04 | 38.10 |
| 1 | host | *Nitraria sibirica* | 106.20 | 38.00 |
| 1 | host | *Nitraria sibirica* | 106.26 | 38.10 |
| 1 | host | *Nitraria sibirica* | 106.30 | 38.30 |
| 1 | host | *Nitraria sibirica* | 106.30 | 38.50 |
| 1 | host | *Nitraria sibirica* | 106.41 | 38.89 |
| 1 | host | *Nitraria sibirica* | 106.60 | 38.90 |
| 1 | host | *Nitraria sibirica* | 106.60 | 37.90 |
| 1 | host | *Nitraria sibirica* | 106.70 | 39.50 |
| 1 | host | *Nitraria sibirica* | 106.99 | 37.74 |
| 1 | host | *Nitraria sibirica* | 107.00 | 40.30 |
| 1 | host | *Nitraria sibirica* | 107.10 | 41.10 |
| 1 | host | *Nitraria sibirica* | 107.30 | 36.60 |
| 1 | host | *Nitraria sibirica* | 107.40 | 40.80 |
| 1 | host | *Nitraria sibirica* | 107.40 | 37.80 |
| 1 | host | *Nitraria sibirica* | 107.52 | 37.10 |
| 1 | host | *Nitraria sibirica* | 107.54 | 37.10 |
| 1 | host | *Nitraria sibirica* | 107.60 | 37.60 |
| 1 | host | *Nitraria sibirica* | 108.00 | 39.10 |
| 1 | host | *Nitraria sibirica* | 108.30 | 41.10 |
| 1 | host | *Nitraria sibirica* | 108.70 | 39.80 |
| 1 | host | *Nitraria sibirica* | 108.72 | 38.59 |
| 1 | host | *Nitraria sibirica* | 108.80 | 37.60 |
| 1 | host | *Nitraria sibirica* | 109.30 | 38.00 |
| 1 | host | *Nitraria sibirica* | 110.00 | 39.80 |
| 1 | host | *Nitraria sibirica* | 110.00 | 37.60 |
| 1 | host | *Nitraria sibirica* | 110.70 | 40.60 |
| 1 | host | *Nitraria sibirica* | 110.70 | 35.60 |
| 1 | host | *Nitraria sibirica* | 110.99 | 40.60 |
| 1 | host | *Nitraria sibirica* | 111.17 | 42.56 |
| 1 | host | *Nitraria sibirica* | 111.80 | 40.90 |
| 1 | host | *Nitraria sibirica* | 112.00 | 43.70 |
| 1 | host | *Nitraria sibirica* | 112.01 | 43.11 |
| 1 | host | *Nitraria sibirica* | 112.60 | 37.70 |
| 1 | host | *Nitraria sibirica* | 112.70 | 42.80 |
| 1 | host | *Nitraria sibirica* | 112.70 | 38.10 |
| 1 | host | *Nitraria sibirica* | 113.10 | 41.00 |
| 1 | host | *Nitraria sibirica* | 113.20 | 40.80 |
| 1 | host | *Nitraria sibirica* | 113.70 | 43.90 |
| 1 | host | *Nitraria sibirica* | 114.20 | 41.10 |
| 1 | host | *Nitraria sibirica* | 114.27 | 40.11 |
| 1 | host | *Nitraria sibirica* | 114.44 | 41.11 |
| 1 | host | *Nitraria sibirica* | 114.80 | 41.30 |
| 1 | host | *Nitraria sibirica* | 114.95 | 44.11 |
| 1 | host | *Nitraria sibirica* | 114.96 | 42.69 |
| 1 | host | *Nitraria sibirica* | 115.00 | 44.00 |
| 1 | host | *Nitraria sibirica* | 115.10 | 40.50 |
| 1 | host | *Nitraria sibirica* | 115.11 | 40.11 |
| 1 | host | *Nitraria sibirica* | 115.34 | 40.11 |
| 1 | host | *Nitraria sibirica* | 116.10 | 44.00 |
| 1 | host | *Nitraria sibirica* | 116.19 | 48.15 |
| 1 | host | *Nitraria sibirica* | 116.40 | 39.90 |
| 1 | host | *Nitraria sibirica* | 116.42 | 48.00 |
| 1 | host | *Nitraria sibirica* | 116.50 | 42.20 |
| 1 | host | *Nitraria sibirica* | 116.52 | 48.58 |
| 1 | host | *Nitraria sibirica* | 116.80 | 48.70 |
| 1 | host | *Nitraria sibirica* | 116.83 | 48.11 |
| 1 | host | *Nitraria sibirica* | 117.00 | 45.50 |
| 1 | host | *Nitraria sibirica* | 117.32 | 49.11 |
| 1 | host | *Nitraria sibirica* | 117.38 | 38.11 |
| 1 | host | *Nitraria sibirica* | 117.39 | 38.11 |
| 1 | host | *Nitraria sibirica* | 117.40 | 38.40 |
| 1 | host | *Nitraria sibirica* | 117.40 | 38.80 |
| 1 | host | *Nitraria sibirica* | 117.50 | 49.60 |
| 1 | host | *Nitraria sibirica* | 117.73 | 39.11 |
| 1 | host | *Nitraria sibirica* | 117.90 | 41.00 |
| 1 | host | *Nitraria sibirica* | 118.00 | 37.40 |
| 1 | host | *Nitraria sibirica* | 118.30 | 48.20 |
| 1 | host | *Nitraria sibirica* | 118.35 | 39.11 |
| 1 | host | *Nitraria sibirica* | 118.36 | 39.11 |
| 1 | host | *Nitraria sibirica* | 118.40 | 39.30 |
| 1 | host | *Nitraria sibirica* | 118.41 | 39.11 |
| 1 | host | *Nitraria sibirica* | 118.70 | 37.40 |
| 1 | host | *Nitraria sibirica* | 119.00 | 42.30 |
| 1 | host | *Nitraria sibirica* | 119.00 | 42.90 |
| 1 | host | *Nitraria sibirica* | 119.20 | 36.70 |
| 1 | host | *Nitraria sibirica* | 119.80 | 49.20 |
| 1 | host | *Nitraria sibirica* | 120.80 | 40.70 |
| 1 | host | *Nitraria sibirica* | 121.53 | 44.67 |
| 1 | host | *Nitraria sibirica* | 122.00 | 41.20 |
| 1 | host | *Nitraria sibirica* | 122.10 | 40.90 |
| 1 | host | *Nitraria sibirica* | 122.30 | 40.60 |
| 1 | host | *Nitraria sibirica* | 122.90 | 45.60 |
| 1 | host | *Nitraria sibirica* | 122.90 | 46.70 |
| 1 | host | *Nitraria sibirica* | 123.10 | 44.80 |
| 1 | host | *Nitraria sibirica* | 123.65 | 41.12 |
| 1 | host | *Nitraria sibirica* | 124.17 | 49.12 |
| 1 | host | *Nitraria sibirica* | 129.40 | 43.10 |
| 2 | parasite | *Boschniakia rossica* | 118.15 | 49.65 |
| 2 | parasite | *Boschniakia rossica* | 119.72 | 49.20 |
| 2 | parasite | *Boschniakia rossica* | 120.12 | 50.21 |
| 2 | parasite | *Boschniakia rossica* | 120.13 | 50.46 |
| 2 | parasite | *Boschniakia rossica* | 120.13 | 50.54 |
| 2 | parasite | *Boschniakia rossica* | 120.18 | 50.25 |
| 2 | parasite | *Boschniakia rossica* | 120.48 | 49.53 |
| 2 | parasite | *Boschniakia rossica* | 121.52 | 50.78 |
| 2 | parasite | *Boschniakia rossica* | 121.77 | 50.90 |
| 2 | parasite | *Boschniakia rossica* | 122.52 | 52.99 |
| 2 | parasite | *Boschniakia rossica* | 122.67 | 52.29 |
| 2 | parasite | *Boschniakia rossica* | 122.81 | 52.93 |
| 2 | parasite | *Boschniakia rossica* | 123.50 | 50.50 |
| 2 | parasite | *Boschniakia rossica* | 123.59 | 52.04 |
| 2 | parasite | *Boschniakia rossica* | 123.66 | 41.87 |
| 2 | parasite | *Boschniakia rossica* | 123.89 | 52.71 |
| 2 | parasite | *Boschniakia rossica* | 124.13 | 50.43 |
| 2 | parasite | *Boschniakia rossica* | 124.39 | 51.67 |
| 2 | parasite | *Boschniakia rossica* | 124.62 | 49.74 |
| 2 | parasite | *Boschniakia rossica* | 124.72 | 52.34 |
| 2 | parasite | *Boschniakia rossica* | 125.41 | 52.44 |
| 2 | parasite | *Boschniakia rossica* | 125.76 | 41.69 |
| 2 | parasite | *Boschniakia rossica* | 125.77 | 52.07 |
| 2 | parasite | *Boschniakia rossica* | 127.40 | 42.19 |
| 2 | parasite | *Boschniakia rossica* | 127.79 | 42.06 |
| 2 | parasite | *Boschniakia rossica* | 127.83 | 41.97 |
| 2 | parasite | *Boschniakia rossica* | 127.96 | 42.04 |
| 2 | parasite | *Boschniakia rossica* | 127.99 | 41.95 |
| 2 | parasite | *Boschniakia rossica* | 128.12 | 42.40 |
| 2 | parasite | *Boschniakia rossica* | 128.15 | 42.46 |
| 2 | parasite | *Boschniakia rossica* | 128.17 | 42.18 |
| 2 | parasite | *Boschniakia rossica* | 128.54 | 45.45 |
| 2 | parasite | *Boschniakia rossica* | 128.91 | 43.12 |
| 2 | parasite | *Boschniakia rossica* | 129.46 | 48.47 |
| 2 | parasite | *Boschniakia rossica* | 129.47 | 42.88 |
| 2 | parasite | *Boschniakia rossica* | 129.54 | 48.49 |
| 2 | parasite | *Boschniakia rossica* | 129.70 | 47.31 |
| 2 | host | *Alnus mandshurica* | 118.20 | 49.68 |
| 2 | host | *Alnus mandshurica* | 119.24 | 48.55 |
| 2 | host | *Alnus mandshurica* | 119.87 | 47.30 |
| 2 | host | *Alnus mandshurica* | 120.85 | 51.95 |
| 2 | host | *Alnus mandshurica* | 121.87 | 51.69 |
| 2 | host | *Alnus mandshurica* | 122.20 | 50.67 |
| 2 | host | *Alnus mandshurica* | 122.36 | 52.96 |
| 2 | host | *Alnus mandshurica* | 122.61 | 51.81 |
| 2 | host | *Alnus mandshurica* | 122.80 | 52.93 |
| 2 | host | *Alnus mandshurica* | 123.17 | 52.74 |
| 2 | host | *Alnus mandshurica* | 123.20 | 53.17 |
| 2 | host | *Alnus mandshurica* | 123.47 | 41.78 |
| 2 | host | *Alnus mandshurica* | 125.15 | 51.12 |
| 2 | host | *Alnus mandshurica* | 125.39 | 41.33 |
| 2 | host | *Alnus mandshurica* | 126.26 | 48.65 |
| 2 | host | *Alnus mandshurica* | 127.70 | 42.23 |
| 2 | host | *Alnus mandshurica* | 127.73 | 42.03 |
| 2 | host | *Alnus mandshurica* | 127.79 | 42.06 |
| 2 | host | *Alnus mandshurica* | 128.00 | 41.97 |
| 2 | host | *Alnus mandshurica* | 128.04 | 42.01 |
| 2 | host | *Alnus mandshurica* | 128.06 | 42.05 |
| 2 | host | *Alnus mandshurica* | 128.06 | 42.08 |
| 2 | host | *Alnus mandshurica* | 128.07 | 42.03 |
| 2 | host | *Alnus mandshurica* | 128.07 | 42.05 |
| 2 | host | *Alnus mandshurica* | 128.07 | 42.07 |
| 2 | host | *Alnus mandshurica* | 128.08 | 42.04 |
| 2 | host | *Alnus mandshurica* | 128.12 | 42.43 |
| 2 | host | *Alnus mandshurica* | 128.13 | 42.13 |
| 2 | host | *Alnus mandshurica* | 128.15 | 42.46 |
| 2 | host | *Alnus mandshurica* | 128.91 | 47.75 |
| 2 | host | *Alnus mandshurica* | 130.44 | 48.83 |
| 2 | host | *Alnus mandshurica* | 130.60 | 48.52 |
| 2 | host | *Alnus mandshurica* | 132.77 | 45.84 |
| 2 | host | *Alnus mandshurica* | 134.54 | 48.33 |
| 3 | parasite | *Cistanche deserticola* | 75.24 | 39.72 |
| 3 | parasite | *Cistanche deserticola* | 75.94 | 39.12 |
| 3 | parasite | *Cistanche deserticola* | 81.13 | 43.82 |
| 3 | parasite | *Cistanche deserticola* | 82.64 | 44.66 |
| 3 | parasite | *Cistanche deserticola* | 83.66 | 44.56 |
| 3 | parasite | *Cistanche deserticola* | 85.16 | 45.29 |
| 3 | parasite | *Cistanche deserticola* | 85.57 | 44.33 |
| 3 | parasite | *Cistanche deserticola* | 85.72 | 46.79 |
| 3 | parasite | *Cistanche deserticola* | 86.28 | 41.75 |
| 3 | parasite | *Cistanche deserticola* | 86.53 | 42.07 |
| 3 | parasite | *Cistanche deserticola* | 87.40 | 43.99 |
| 3 | parasite | *Cistanche deserticola* | 87.48 | 47.10 |
| 3 | parasite | *Cistanche deserticola* | 87.57 | 43.81 |
| 3 | parasite | *Cistanche deserticola* | 87.58 | 43.80 |
| 3 | parasite | *Cistanche deserticola* | 87.61 | 43.66 |
| 3 | parasite | *Cistanche deserticola* | 87.72 | 43.58 |
| 3 | parasite | *Cistanche deserticola* | 87.89 | 43.55 |
| 3 | parasite | *Cistanche deserticola* | 88.07 | 43.41 |
| 3 | parasite | *Cistanche deserticola* | 88.29 | 43.36 |
| 3 | parasite | *Cistanche deserticola* | 89.51 | 46.99 |
| 3 | parasite | *Cistanche deserticola* | 89.53 | 47.01 |
| 3 | parasite | *Cistanche deserticola* | 91.60 | 37.54 |
| 3 | parasite | *Cistanche deserticola* | 97.12 | 41.90 |
| 3 | parasite | *Cistanche deserticola* | 98.48 | 39.67 |
| 3 | parasite | *Cistanche deserticola* | 103.24 | 40.95 |
| 3 | parasite | *Cistanche deserticola* | 104.92 | 37.60 |
| 3 | parasite | *Cistanche deserticola* | 105.55 | 38.79 |
| 3 | parasite | *Cistanche deserticola* | 105.65 | 38.85 |
| 3 | parasite | *Cistanche deserticola* | 105.70 | 39.73 |
| 3 | parasite | *Cistanche deserticola* | 105.89 | 38.87 |
| 3 | parasite | *Cistanche deserticola* | 106.63 | 42.16 |
| 3 | parasite | *Cistanche deserticola* | 107.05 | 40.34 |
| 3 | parasite | *Cistanche deserticola* | 107.37 | 40.79 |
| 3 | host | *Haloxylon ammodendron* | 76.00 | 39.50 |
| 3 | host | *Haloxylon ammodendron* | 76.20 | 39.70 |
| 3 | host | *Haloxylon ammodendron* | 76.20 | 38.90 |
| 3 | host | *Haloxylon ammodendron* | 76.70 | 39.50 |
| 3 | host | *Haloxylon ammodendron* | 77.40 | 37.90 |
| 3 | host | *Haloxylon ammodendron* | 77.60 | 38.90 |
| 3 | host | *Haloxylon ammodendron* | 78.60 | 39.80 |
| 3 | host | *Haloxylon ammodendron* | 79.10 | 40.50 |
| 3 | host | *Haloxylon ammodendron* | 79.30 | 36.20 |
| 3 | host | *Haloxylon ammodendron* | 80.20 | 41.10 |
| 3 | host | *Haloxylon ammodendron* | 80.90 | 44.10 |
| 3 | host | *Haloxylon ammodendron* | 81.20 | 43.80 |
| 3 | host | *Haloxylon ammodendron* | 81.30 | 40.60 |
| 3 | host | *Haloxylon ammodendron* | 81.50 | 44.00 |
| 3 | host | *Haloxylon ammodendron* | 81.75 | 44.08 |
| 3 | host | *Haloxylon ammodendron* | 81.90 | 41.80 |
| 3 | host | *Haloxylon ammodendron* | 82.10 | 44.90 |
| 3 | host | *Haloxylon ammodendron* | 82.60 | 45.20 |
| 3 | host | *Haloxylon ammodendron* | 82.80 | 41.20 |
| 3 | host | *Haloxylon ammodendron* | 82.90 | 44.60 |
| 3 | host | *Haloxylon ammodendron* | 82.93 | 44.69 |
| 3 | host | *Haloxylon ammodendron* | 83.00 | 41.70 |
| 3 | host | *Haloxylon ammodendron* | 83.00 | 46.80 |
| 3 | host | *Haloxylon ammodendron* | 83.06 | 42.12 |
| 3 | host | *Haloxylon ammodendron* | 83.07 | 42.12 |
| 3 | host | *Haloxylon ammodendron* | 83.60 | 45.90 |
| 3 | host | *Haloxylon ammodendron* | 83.60 | 46.50 |
| 3 | host | *Haloxylon ammodendron* | 84.25 | 41.78 |
| 3 | host | *Haloxylon ammodendron* | 84.30 | 41.80 |
| 3 | host | *Haloxylon ammodendron* | 84.70 | 44.40 |
| 3 | host | *Haloxylon ammodendron* | 84.77 | 44.18 |
| 3 | host | *Haloxylon ammodendron* | 84.78 | 44.32 |
| 3 | host | *Haloxylon ammodendron* | 84.90 | 45.60 |
| 3 | host | *Haloxylon ammodendron* | 84.90 | 44.40 |
| 3 | host | *Haloxylon ammodendron* | 84.97 | 43.43 |
| 3 | host | *Haloxylon ammodendron* | 85.30 | 42.22 |
| 3 | host | *Haloxylon ammodendron* | 85.50 | 38.20 |
| 3 | host | *Haloxylon ammodendron* | 85.60 | 44.30 |
| 3 | host | *Haloxylon ammodendron* | 85.70 | 46.10 |
| 3 | host | *Haloxylon ammodendron* | 85.70 | 46.80 |
| 3 | host | *Haloxylon ammodendron* | 86.00 | 44.30 |
| 3 | host | *Haloxylon ammodendron* | 86.20 | 41.90 |
| 3 | host | *Haloxylon ammodendron* | 86.22 | 44.95 |
| 3 | host | *Haloxylon ammodendron* | 86.24 | 45.05 |
| 3 | host | *Haloxylon ammodendron* | 86.30 | 41.30 |
| 3 | host | *Haloxylon ammodendron* | 86.30 | 47.50 |
| 3 | host | *Haloxylon ammodendron* | 86.30 | 44.30 |
| 3 | host | *Haloxylon ammodendron* | 86.52 | 45.43 |
| 3 | host | *Haloxylon ammodendron* | 86.60 | 42.10 |
| 3 | host | *Haloxylon ammodendron* | 86.90 | 42.30 |
| 3 | host | *Haloxylon ammodendron* | 86.90 | 44.20 |
| 3 | host | *Haloxylon ammodendron* | 87.30 | 48.30 |
| 3 | host | *Haloxylon ammodendron* | 87.50 | 44.20 |
| 3 | host | *Haloxylon ammodendron* | 87.70 | 44.00 |
| 3 | host | *Haloxylon ammodendron* | 87.80 | 47.90 |
| 3 | host | *Haloxylon ammodendron* | 87.93 | 44.28 |
| 3 | host | *Haloxylon ammodendron* | 88.00 | 44.00 |
| 3 | host | *Haloxylon ammodendron* | 88.00 | 46.60 |
| 3 | host | *Haloxylon ammodendron* | 88.13 | 44.34 |
| 3 | host | *Haloxylon ammodendron* | 88.20 | 39.00 |
| 3 | host | *Haloxylon ammodendron* | 88.29 | 44.67 |
| 3 | host | *Haloxylon ammodendron* | 88.30 | 43.40 |
| 3 | host | *Haloxylon ammodendron* | 88.70 | 42.80 |
| 3 | host | *Haloxylon ammodendron* | 88.83 | 44.55 |
| 3 | host | *Haloxylon ammodendron* | 88.99 | 44.64 |
| 3 | host | *Haloxylon ammodendron* | 89.16 | 42.87 |
| 3 | host | *Haloxylon ammodendron* | 89.20 | 42.87 |
| 3 | host | *Haloxylon ammodendron* | 89.20 | 44.00 |
| 3 | host | *Haloxylon ammodendron* | 89.20 | 43.00 |
| 3 | host | *Haloxylon ammodendron* | 89.30 | 42.87 |
| 3 | host | *Haloxylon ammodendron* | 89.50 | 46.50 |
| 3 | host | *Haloxylon ammodendron* | 89.60 | 44.00 |
| 3 | host | *Haloxylon ammodendron* | 90.06 | 44.22 |
| 3 | host | *Haloxylon ammodendron* | 90.08 | 45.07 |
| 3 | host | *Haloxylon ammodendron* | 90.20 | 42.90 |
| 3 | host | *Haloxylon ammodendron* | 90.30 | 43.80 |
| 3 | host | *Haloxylon ammodendron* | 90.40 | 46.20 |
| 3 | host | *Haloxylon ammodendron* | 93.00 | 44.20 |
| 3 | host | *Haloxylon ammodendron* | 93.30 | 38.10 |
| 3 | host | *Haloxylon ammodendron* | 93.50 | 42.80 |
| 3 | host | *Haloxylon ammodendron* | 94.30 | 39.60 |
| 3 | host | *Haloxylon ammodendron* | 94.69 | 40.09 |
| 3 | host | *Haloxylon ammodendron* | 94.70 | 40.10 |
| 3 | host | *Haloxylon ammodendron* | 94.70 | 43.30 |
| 3 | host | *Haloxylon ammodendron* | 94.90 | 39.50 |
| 3 | host | *Haloxylon ammodendron* | 94.90 | 36.40 |
| 3 | host | *Haloxylon ammodendron* | 95.75 | 41.95 |
| 3 | host | *Haloxylon ammodendron* | 95.78 | 40.55 |
| 3 | host | *Haloxylon ammodendron* | 95.80 | 40.50 |
| 3 | host | *Haloxylon ammodendron* | 97.10 | 40.30 |
| 3 | host | *Haloxylon ammodendron* | 97.40 | 37.40 |
| 3 | host | *Haloxylon ammodendron* | 97.60 | 36.21 |
| 3 | host | *Haloxylon ammodendron* | 98.10 | 36.30 |
| 3 | host | *Haloxylon ammodendron* | 98.50 | 36.90 |
| 3 | host | *Haloxylon ammodendron* | 98.70 | 40.28 |
| 3 | host | *Haloxylon ammodendron* | 98.71 | 40.28 |
| 3 | host | *Haloxylon ammodendron* | 98.90 | 40.00 |
| 3 | host | *Haloxylon ammodendron* | 99.80 | 39.40 |
| 3 | host | *Haloxylon ammodendron* | 100.20 | 39.20 |
| 3 | host | *Haloxylon ammodendron* | 101.10 | 42.00 |
| 3 | host | *Haloxylon ammodendron* | 101.20 | 36.60 |
| 3 | host | *Haloxylon ammodendron* | 101.70 | 39.20 |
| 3 | host | *Haloxylon ammodendron* | 102.00 | 38.30 |
| 3 | host | *Haloxylon ammodendron* | 102.60 | 37.90 |
| 3 | host | *Haloxylon ammodendron* | 102.71 | 40.44 |
| 3 | host | *Haloxylon ammodendron* | 102.90 | 37.50 |
| 3 | host | *Haloxylon ammodendron* | 103.10 | 38.60 |
| 3 | host | *Haloxylon ammodendron* | 103.12 | 38.62 |
| 3 | host | *Haloxylon ammodendron* | 103.31 | 39.02 |
| 3 | host | *Haloxylon ammodendron* | 103.61 | 39.07 |
| 3 | host | *Haloxylon ammodendron* | 103.64 | 38.95 |
| 3 | host | *Haloxylon ammodendron* | 103.80 | 36.10 |
| 3 | host | *Haloxylon ammodendron* | 103.85 | 38.63 |
| 3 | host | *Haloxylon ammodendron* | 104.90 | 34.70 |
| 3 | host | *Haloxylon ammodendron* | 105.01 | 37.48 |
| 3 | host | *Haloxylon ammodendron* | 105.20 | 37.50 |
| 3 | host | *Haloxylon ammodendron* | 105.70 | 38.90 |
| 3 | host | *Haloxylon ammodendron* | 106.63 | 42.16 |
| 3 | host | *Haloxylon ammodendron* | 106.80 | 39.70 |
| 3 | host | *Haloxylon ammodendron* | 107.00 | 40.30 |
| 3 | host | *Haloxylon ammodendron* | 107.06 | 40.35 |
| 3 | host | *Haloxylon ammodendron* | 107.10 | 41.10 |
| 3 | host | *Haloxylon ammodendron* | 107.40 | 40.80 |
| 3 | host | *Haloxylon ammodendron* | 107.40 | 37.80 |
| 3 | host | *Haloxylon ammodendron* | 108.30 | 41.10 |
| 3 | host | *Haloxylon ammodendron* | 108.70 | 39.80 |
| 3 | host | *Haloxylon ammodendron* | 108.90 | 38.60 |
| 3 | host | *Haloxylon ammodendron* | 109.75 | 40.51 |
| 3 | host | *Haloxylon ammodendron* | 110.00 | 39.80 |
| 3 | host | *Haloxylon ammodendron* | 112.00 | 43.70 |
| 4 | parasite | *Cistanche mongolica* | 76.38 | 39.09 |
| 4 | parasite | *Cistanche mongolica* | 77.26 | 38.42 |
| 4 | parasite | *Cistanche mongolica* | 77.35 | 38.42 |
| 4 | parasite | *Cistanche mongolica* | 77.58 | 37.87 |
| 4 | parasite | *Cistanche mongolica* | 77.74 | 38.97 |
| 4 | parasite | *Cistanche mongolica* | 77.77 | 38.33 |
| 4 | parasite | *Cistanche mongolica* | 78.28 | 37.57 |
| 4 | parasite | *Cistanche mongolica* | 78.52 | 39.78 |
| 4 | parasite | *Cistanche mongolica* | 78.55 | 39.80 |
| 4 | parasite | *Cistanche mongolica* | 79.71 | 37.26 |
| 4 | parasite | *Cistanche mongolica* | 79.73 | 37.28 |
| 4 | parasite | *Cistanche mongolica* | 79.82 | 37.08 |
| 4 | parasite | *Cistanche mongolica* | 79.84 | 37.30 |
| 4 | parasite | *Cistanche mongolica* | 79.92 | 37.12 |
| 4 | parasite | *Cistanche mongolica* | 80.33 | 40.24 |
| 4 | parasite | *Cistanche mongolica* | 80.37 | 40.65 |
| 4 | parasite | *Cistanche mongolica* | 80.40 | 40.65 |
| 4 | parasite | *Cistanche mongolica* | 80.48 | 37.09 |
| 4 | parasite | *Cistanche mongolica* | 80.73 | 37.02 |
| 4 | parasite | *Cistanche mongolica* | 80.82 | 36.97 |
| 4 | parasite | *Cistanche mongolica* | 81.69 | 36.87 |
| 4 | parasite | *Cistanche mongolica* | 81.87 | 36.84 |
| 4 | parasite | *Cistanche mongolica* | 81.89 | 36.81 |
| 4 | parasite | *Cistanche mongolica* | 82.01 | 40.71 |
| 4 | parasite | *Cistanche mongolica* | 82.19 | 36.90 |
| 4 | parasite | *Cistanche mongolica* | 82.35 | 36.54 |
| 4 | parasite | *Cistanche mongolica* | 82.55 | 37.93 |
| 4 | parasite | *Cistanche mongolica* | 82.92 | 37.18 |
| 4 | parasite | *Cistanche mongolica* | 82.95 | 37.15 |
| 4 | parasite | *Cistanche mongolica* | 82.97 | 37.12 |
| 4 | parasite | *Cistanche mongolica* | 82.99 | 37.12 |
| 4 | parasite | *Cistanche mongolica* | 83.20 | 37.91 |
| 4 | parasite | *Cistanche mongolica* | 83.27 | 37.23 |
| 4 | parasite | *Cistanche mongolica* | 83.52 | 37.98 |
| 4 | parasite | *Cistanche mongolica* | 83.67 | 37.93 |
| 4 | parasite | *Cistanche mongolica* | 83.67 | 37.94 |
| 4 | parasite | *Cistanche mongolica* | 84.03 | 41.08 |
| 4 | parasite | *Cistanche mongolica* | 84.16 | 37.69 |
| 4 | parasite | *Cistanche mongolica* | 84.23 | 41.78 |
| 4 | parasite | *Cistanche mongolica* | 84.25 | 41.78 |
| 4 | parasite | *Cistanche mongolica* | 85.53 | 38.15 |
| 4 | parasite | *Cistanche mongolica* | 85.54 | 38.12 |
| 4 | parasite | *Cistanche mongolica* | 85.64 | 37.78 |
| 4 | parasite | *Cistanche mongolica* | 86.07 | 37.71 |
| 4 | parasite | *Cistanche mongolica* | 87.07 | 40.83 |
| 4 | parasite | *Cistanche mongolica* | 88.75 | 38.96 |
| 4 | host | *Tamarix ramosissima* | 75.30 | 39.70 |
| 4 | host | *Tamarix ramosissima* | 75.90 | 39.40 |
| 4 | host | *Tamarix ramosissima* | 76.00 | 39.20 |
| 4 | host | *Tamarix ramosissima* | 76.10 | 39.40 |
| 4 | host | *Tamarix ramosissima* | 76.20 | 38.90 |
| 4 | host | *Tamarix ramosissima* | 76.20 | 39.70 |
| 4 | host | *Tamarix ramosissima* | 76.68 | 39.85 |
| 4 | host | *Tamarix ramosissima* | 76.70 | 39.50 |
| 4 | host | *Tamarix ramosissima* | 76.80 | 39.20 |
| 4 | host | *Tamarix ramosissima* | 77.30 | 38.40 |
| 4 | host | *Tamarix ramosissima* | 77.40 | 37.90 |
| 4 | host | *Tamarix ramosissima* | 77.60 | 38.90 |
| 4 | host | *Tamarix ramosissima* | 78.30 | 37.60 |
| 4 | host | *Tamarix ramosissima* | 78.60 | 39.80 |
| 4 | host | *Tamarix ramosissima* | 79.30 | 36.20 |
| 4 | host | *Tamarix ramosissima* | 80.20 | 41.10 |
| 4 | host | *Tamarix ramosissima* | 80.20 | 41.30 |
| 4 | host | *Tamarix ramosissima* | 80.80 | 37.00 |
| 4 | host | *Tamarix ramosissima* | 80.90 | 44.10 |
| 4 | host | *Tamarix ramosissima* | 81.20 | 43.80 |
| 4 | host | *Tamarix ramosissima* | 81.70 | 36.90 |
| 4 | host | *Tamarix ramosissima* | 82.00 | 42.68 |
| 4 | host | *Tamarix ramosissima* | 82.10 | 44.90 |
| 4 | host | *Tamarix ramosissima* | 82.20 | 43.50 |
| 4 | host | *Tamarix ramosissima* | 82.60 | 45.20 |
| 4 | host | *Tamarix ramosissima* | 82.70 | 37.10 |
| 4 | host | *Tamarix ramosissima* | 82.80 | 41.20 |
| 4 | host | *Tamarix ramosissima* | 82.90 | 44.60 |
| 4 | host | *Tamarix ramosissima* | 83.00 | 41.70 |
| 4 | host | *Tamarix ramosissima* | 84.22 | 41.26 |
| 4 | host | *Tamarix ramosissima* | 84.30 | 41.80 |
| 4 | host | *Tamarix ramosissima* | 84.70 | 44.40 |
| 4 | host | *Tamarix ramosissima* | 84.90 | 44.40 |
| 4 | host | *Tamarix ramosissima* | 84.90 | 45.60 |
| 4 | host | *Tamarix ramosissima* | 85.10 | 45.70 |
| 4 | host | *Tamarix ramosissima* | 85.50 | 38.20 |
| 4 | host | *Tamarix ramosissima* | 85.60 | 44.30 |
| 4 | host | *Tamarix ramosissima* | 85.70 | 46.80 |
| 4 | host | *Tamarix ramosissima* | 86.00 | 44.30 |
| 4 | host | *Tamarix ramosissima* | 86.20 | 41.80 |
| 4 | host | *Tamarix ramosissima* | 86.30 | 41.30 |
| 4 | host | *Tamarix ramosissima* | 86.30 | 44.30 |
| 4 | host | *Tamarix ramosissima* | 86.40 | 42.30 |
| 4 | host | *Tamarix ramosissima* | 86.40 | 48.40 |
| 4 | host | *Tamarix ramosissima* | 86.60 | 42.10 |
| 4 | host | *Tamarix ramosissima* | 86.90 | 42.30 |
| 4 | host | *Tamarix ramosissima* | 86.90 | 44.20 |
| 4 | host | *Tamarix ramosissima* | 87.30 | 44.00 |
| 4 | host | *Tamarix ramosissima* | 87.30 | 48.30 |
| 4 | host | *Tamarix ramosissima* | 87.35 | 43.33 |
| 4 | host | *Tamarix ramosissima* | 87.50 | 43.40 |
| 4 | host | *Tamarix ramosissima* | 87.58 | 43.81 |
| 4 | host | *Tamarix ramosissima* | 87.60 | 43.80 |
| 4 | host | *Tamarix ramosissima* | 87.62 | 43.83 |
| 4 | host | *Tamarix ramosissima* | 87.80 | 47.30 |
| 4 | host | *Tamarix ramosissima* | 87.80 | 47.90 |
| 4 | host | *Tamarix ramosissima* | 87.86 | 44.30 |
| 4 | host | *Tamarix ramosissima* | 87.93 | 44.28 |
| 4 | host | *Tamarix ramosissima* | 88.00 | 44.00 |
| 4 | host | *Tamarix ramosissima* | 88.20 | 39.00 |
| 4 | host | *Tamarix ramosissima* | 88.30 | 43.40 |
| 4 | host | *Tamarix ramosissima* | 88.70 | 42.80 |
| 4 | host | *Tamarix ramosissima* | 89.20 | 43.00 |
| 4 | host | *Tamarix ramosissima* | 89.20 | 44.00 |
| 4 | host | *Tamarix ramosissima* | 89.50 | 46.50 |
| 4 | host | *Tamarix ramosissima* | 89.60 | 44.00 |
| 4 | host | *Tamarix ramosissima* | 90.20 | 42.90 |
| 4 | host | *Tamarix ramosissima* | 90.40 | 46.20 |
| 4 | host | *Tamarix ramosissima* | 93.00 | 44.20 |
| 4 | host | *Tamarix ramosissima* | 93.30 | 38.10 |
| 4 | host | *Tamarix ramosissima* | 93.50 | 42.80 |
| 4 | host | *Tamarix ramosissima* | 93.70 | 43.33 |
| 4 | host | *Tamarix ramosissima* | 94.36 | 39.63 |
| 4 | host | *Tamarix ramosissima* | 94.70 | 40.10 |
| 4 | host | *Tamarix ramosissima* | 94.90 | 36.40 |
| 4 | host | *Tamarix ramosissima* | 94.90 | 39.50 |
| 4 | host | *Tamarix ramosissima* | 95.80 | 40.50 |
| 4 | host | *Tamarix ramosissima* | 97.10 | 40.30 |
| 4 | host | *Tamarix ramosissima* | 97.40 | 37.40 |
| 4 | host | *Tamarix ramosissima* | 98.10 | 36.30 |
| 4 | host | *Tamarix ramosissima* | 98.30 | 39.80 |
| 4 | host | *Tamarix ramosissima* | 98.35 | 39.83 |
| 4 | host | *Tamarix ramosissima* | 98.50 | 39.70 |
| 4 | host | *Tamarix ramosissima* | 98.72 | 40.27 |
| 4 | host | *Tamarix ramosissima* | 98.73 | 40.27 |
| 4 | host | *Tamarix ramosissima* | 98.73 | 40.28 |
| 4 | host | *Tamarix ramosissima* | 98.90 | 40.00 |
| 4 | host | *Tamarix ramosissima* | 99.08 | 36.77 |
| 4 | host | *Tamarix ramosissima* | 99.60 | 35.60 |
| 4 | host | *Tamarix ramosissima* | 99.60 | 38.80 |
| 4 | host | *Tamarix ramosissima* | 100.00 | 36.40 |
| 4 | host | *Tamarix ramosissima* | 100.20 | 39.20 |
| 4 | host | *Tamarix ramosissima* | 100.50 | 38.90 |
| 4 | host | *Tamarix ramosissima* | 100.80 | 38.40 |
| 4 | host | *Tamarix ramosissima* | 101.09 | 41.96 |
| 4 | host | *Tamarix ramosissima* | 101.10 | 38.80 |
| 4 | host | *Tamarix ramosissima* | 101.10 | 42.00 |
| 4 | host | *Tamarix ramosissima* | 101.13 | 41.99 |
| 4 | host | *Tamarix ramosissima* | 101.50 | 35.90 |
| 4 | host | *Tamarix ramosissima* | 101.70 | 39.20 |
| 4 | host | *Tamarix ramosissima* | 101.79 | 36.65 |
| 4 | host | *Tamarix ramosissima* | 102.00 | 36.40 |
| 4 | host | *Tamarix ramosissima* | 102.20 | 36.90 |
| 4 | host | *Tamarix ramosissima* | 103.10 | 38.60 |
| 4 | host | *Tamarix ramosissima* | 103.26 | 39.04 |
| 4 | host | *Tamarix ramosissima* | 103.28 | 39.03 |
| 4 | host | *Tamarix ramosissima* | 103.61 | 39.07 |
| 4 | host | *Tamarix ramosissima* | 103.80 | 36.10 |
| 4 | host | *Tamarix ramosissima* | 103.80 | 37.23 |
| 4 | host | *Tamarix ramosissima* | 104.01 | 36.16 |
| 4 | host | *Tamarix ramosissima* | 104.02 | 36.23 |
| 4 | host | *Tamarix ramosissima* | 104.10 | 37.20 |
| 4 | host | *Tamarix ramosissima* | 104.18 | 37.24 |
| 4 | host | *Tamarix ramosissima* | 104.22 | 36.15 |
| 4 | host | *Tamarix ramosissima* | 105.70 | 38.80 |
| 4 | host | *Tamarix ramosissima* | 105.90 | 37.00 |
| 4 | host | *Tamarix ramosissima* | 105.93 | 38.02 |
| 4 | host | *Tamarix ramosissima* | 106.20 | 38.50 |
| 4 | host | *Tamarix ramosissima* | 106.80 | 39.70 |
| 4 | host | *Tamarix ramosissima* | 107.00 | 40.30 |
| 4 | host | *Tamarix ramosissima* | 107.10 | 41.10 |
| 4 | host | *Tamarix ramosissima* | 107.20 | 40.90 |
| 4 | host | *Tamarix ramosissima* | 107.40 | 40.80 |
| 4 | host | *Tamarix ramosissima* | 107.44 | 40.75 |
| 4 | host | *Tamarix ramosissima* | 108.00 | 33.50 |
| 4 | host | *Tamarix ramosissima* | 108.30 | 41.10 |
| 4 | host | *Tamarix ramosissima* | 108.70 | 39.80 |
| 4 | host | *Tamarix ramosissima* | 108.80 | 37.60 |
| 4 | host | *Tamarix ramosissima* | 108.90 | 38.60 |
| 4 | host | *Tamarix ramosissima* | 109.30 | 36.90 |
| 4 | host | *Tamarix ramosissima* | 109.70 | 38.30 |
| 4 | host | *Tamarix ramosissima* | 109.90 | 40.60 |
| 4 | host | *Tamarix ramosissima* | 110.00 | 39.80 |
| 4 | host | *Tamarix ramosissima* | 110.30 | 37.50 |
| 4 | host | *Tamarix ramosissima* | 110.50 | 38.80 |
| 4 | host | *Tamarix ramosissima* | 111.00 | 38.00 |
| 4 | host | *Tamarix ramosissima* | 111.10 | 39.40 |
| 4 | host | *Tamarix ramosissima* | 111.20 | 37.90 |
| 4 | host | *Tamarix ramosissima* | 111.20 | 39.90 |
| 4 | host | *Tamarix ramosissima* | 111.70 | 35.30 |
| 4 | host | *Tamarix ramosissima* | 111.70 | 39.90 |
| 4 | host | *Tamarix ramosissima* | 111.70 | 40.80 |
| 4 | host | *Tamarix ramosissima* | 112.00 | 34.00 |
| 4 | host | *Tamarix ramosissima* | 112.40 | 37.60 |
| 4 | host | *Tamarix ramosissima* | 112.60 | 40.90 |
| 4 | host | *Tamarix ramosissima* | 112.90 | 35.50 |
| 4 | host | *Tamarix ramosissima* | 113.91 | 33.83 |
| 4 | host | *Tamarix ramosissima* | 114.10 | 26.60 |
| 4 | host | *Tamarix ramosissima* | 114.50 | 38.10 |
| 4 | host | *Tamarix ramosissima* | 114.60 | 34.70 |
| 4 | host | *Tamarix ramosissima* | 114.80 | 41.30 |
| 4 | host | *Tamarix ramosissima* | 116.20 | 40.00 |
| 4 | host | *Tamarix ramosissima* | 116.40 | 39.90 |
| 4 | host | *Tamarix ramosissima* | 116.60 | 35.40 |
| 4 | host | *Tamarix ramosissima* | 116.90 | 34.00 |
| 4 | host | *Tamarix ramosissima* | 117.00 | 36.70 |
| 4 | host | *Tamarix ramosissima* | 117.10 | 34.80 |
| 4 | host | *Tamarix ramosissima* | 117.30 | 39.10 |
| 4 | host | *Tamarix ramosissima* | 117.40 | 38.80 |
| 4 | host | *Tamarix ramosissima* | 118.20 | 41.00 |
| 4 | host | *Tamarix ramosissima* | 118.70 | 37.40 |
| 4 | host | *Tamarix ramosissima* | 118.70 | 43.50 |
| 4 | host | *Tamarix ramosissima* | 118.80 | 37.70 |
| 4 | host | *Tamarix ramosissima* | 119.00 | 42.30 |
| 4 | host | *Tamarix ramosissima* | 122.00 | 46.10 |
| 4 | host | *Tamarix ramosissima* | 122.30 | 43.70 |
| 4 | host | *Tamarix ramosissima* | 124.80 | 46.00 |
| 4 | host | *Tamarix ramosissima* | 126.70 | 46.00 |
