# Supplementary material for: Predicting the potential distribution of four endangered holoparasites and their primary hosts in China under climate change
Source: Front Plant Sci. 2022 Aug 3;13:942448. doi: 10.3389/fpls.2022.942448 (PMC9384867; doi:10.3389/fpls.2022.942448)
Supplement: Supplementary file 2 [file Table_2.DOCX]

**Supplementary Table 2**

Classification and representation of 19 bioclimatic factors.

| **Classification** | **Code** | **Explanation** |
| --- | --- | --- |
| **Temperature factors** | **Bio1** | Annual mean temperature |
|  | **Bio2** | Mean diurnal range |
|  | **Bio3** | Isothermality |
|  | **Bio4** | Temperature seasonality |
|  | **Bio5** | Max temperature of warmest month |
|  | **Bio6** | Min temperature of coldest month |
|  | **Bio7** | Temperature annual range |
|  | **Bio8** | Mean temperature of wettest quarter |
|  | **Bio9** | Mean temperature of driest quarter |
|  | **Bio10** | Mean temperature of warmest quarter |
|  | **Bio11** | Mean temperature of coldest quarter |
| **Precipitation factors** | **Bio12** | Annual precipitation |
|  | **Bio13** | Precipitation of wettest month |
|  | **Bio14** | Precipitation of driest month |
|  | **Bio15** | Precipitation seasonality |
|  | **Bio16** | Precipitation of wettest quarter |
|  | **Bio17** | Precipitation of driest quarter |
|  | **Bio18** | Precipitation of warmest quarter |
|  | **Bio19** | Precipitation of coldest quarter |
