# Supplementary material for: Predicting the potential distribution of four endangered holoparasites and their primary hosts in China under climate change
Source: Front Plant Sci. 2022 Aug 3;13:942448. doi: 10.3389/fpls.2022.942448 (PMC9384867; doi:10.3389/fpls.2022.942448)
Supplement: Supplementary file 3 [file Table_3.DOCX]

**Supplementary Table 3**

Percent contribution of the climatic factors included in the MaxEnt model of four holoparasitic plants and their primary hosts.

| **No.** | **Species** | **Climatic factors** | **Percent contribution (%)** |
| --- | --- | --- | --- |
| 1 | P: *Cynomorium songaricum* | Bio16 | 36.5 |
|  |  | Bio2 | 17.7 |
|  |  | Bio11 | 16.9 |
|  |  | Bio7 | 14.2 |
|  |  | Bio17 | 11.6 |
|  |  | Bio15 | 3.0 |
|  | H: *Nitraria sibirica* | Bio7 | 38.6 |
|  |  | Bio17 | 22.1 |
|  |  | Bio6 | 18.7 |
|  |  | Bio12 | 7.4 |
|  |  | Bio5 | 6.0 |
|  |  | Bio15 | 5.1 |
|  |  | Bio2 | 2.2 |
| 2 | P: *Boschniakia rossica* | Bio4 | 71.1 |
|  |  | Bio10 | 13.2 |
|  |  | Bio14 | 12.9 |
|  |  | Bio3 | 2.8 |
|  |  | Bio8 | 0.0 |
|  | H: *Alnus mandshurica* | Bio4 | 34.5 |
|  |  | Bio9 | 31.0 |
|  |  | Bio14 | 25.3 |
|  |  | Bio10 | 7.7 |
|  |  | Bio1 | 1.4 |
| 3 | P: *Cistanche deserticola* | Bio18 | 48.9 |
|  |  | Bio15 | 13.7 |
|  |  | Bio12 | 13.2 |
|  |  | Bio4 | 11.2 |
|  |  | Bio6 | 6.4 |
|  |  | Bio5 | 3.5 |
|  |  | Bio2 | 3.1 |
|  | H: *Haloxylon ammodendron* | Bio13 | 54.3 |
|  |  | Bio4 | 18.7 |
|  |  | Bio15 | 8.9 |
|  |  | Bio19 | 6.9 |
|  |  | Bio1 | 6.0 |
|  |  | Bio2 | 5.1 |
| 4 | P: *Cistanche mongolica* | Bio18 | 53.2 |
|  |  | Bio11 | 23.8 |
|  |  | Bio14 | 12.5 |
|  |  | Bio8 | 6.9 |
|  |  | Bio4 | 2.8 |
|  |  | Bio17 | 0.4 |
|  |  | Bio2 | 0.4 |
|  | H: *Tamarix ramosissima* | Bio4 | 40.3 |
|  |  | Bio6 | 24.9 |
|  |  | Bio18 | 15.4 |
|  |  | Bio2 | 8.9 |
|  |  | Bio17 | 6.8 |
|  |  | Bio15 | 2.4 |
|  |  | Bio5 | 1.3 |

* P: parasite; H: host.
