# Supplementary material for: Predicting the potential distribution of four endangered holoparasites and their primary hosts in China under climate change
Source: Front Plant Sci. 2022 Aug 3;13:942448. doi: 10.3389/fpls.2022.942448 (PMC9384867; doi:10.3389/fpls.2022.942448)
Supplement: Supplementary file 4 [file Table_4.DOCX]

**Supplementary Table 4**

Feature class combination and regularization multiplier included in the MaxEnt model of four holoparasitic plants and their primary hosts.

| **No.** | **Species** | **Feature class combination** | **Regularization multiplier** |
| --- | --- | --- | --- |
| 1 | P: *Cynomorium songaricum* | LQHP | 0.5 |
|  | H: *Nitraria sibirica* | LQH | 1.0 |
| 2 | P: *Boschniakia rossica* | LQH | 2.5 |
|  | H: *Alnus mandshurica* | LQH | 0.5 |
| 3 | P: *Cistanche deserticola* | LQH | 1.0 |
|  | H: *Haloxylon ammodendron* | LQHP | 1.5 |
| 4 | P: *Cistanche mongolica* | LQH | 1.0 |
|  | H: *Tamarix ramosissima* | LQHP | 2.0 |

* P: parasite; H: host.
